# KDM4A regulates myogenesis by demethylating H3K9me3 of myogenic regulatory factors

**DOI:** 10.1038/s41419-021-03799-1

**Published:** 2021-05-19

**Authors:** Qi Zhu, Feng Liang, Shufang Cai, Xiaorong Luo, Tianqi Duo, Ziyun Liang, Zuyong He, Yaosheng Chen, Delin Mo

**Affiliations:** State Key Laboratory of Biocontrol, School of Life Sciences, Sun Yat-sen University, North Third Road, Higher Education Mega Center, 510006 Guangzhou, Guangdong China

**Keywords:** Differentiation, Muscle stem cells, Epigenetics

## Abstract

Histone lysine demethylase 4A (KDM4A) plays a crucial role in regulating cell proliferation, cell differentiation, development and tumorigenesis. However, little is known about the function of KDM4A in muscle development and regeneration. Here, we found that the conditional ablation of KDM4A in skeletal muscle caused impairment of embryonic and postnatal muscle formation. The loss of KDM4A in satellite cells led to defective muscle regeneration and blocked the proliferation and differentiation of satellite cells. Myogenic differentiation and myotube formation in KDM4A-deficient myoblasts were inhibited. Chromatin immunoprecipitation assay revealed that KDM4A promoted myogenesis by removing the histone methylation mark H3K9me3 at MyoD, MyoG and Myf5 locus. Furthermore, inactivation of KDM4A in myoblasts suppressed myoblast differentiation and accelerated H3K9me3 level. Knockdown of KDM4A in vitro reduced myoblast proliferation through enhancing the expression of the cyclin-dependent kinase inhibitor P21 and decreasing the expression of cell cycle regulator Cyclin D1. Together, our findings identify KDM4A as an important regulator for skeletal muscle development and regeneration, orchestrating myogenic cell proliferation and differentiation.

## Introduction

Skeletal muscle is an important tissue of mammalian body orchestrating metabolism and homeostasis^1^, which has a robust capacity of regeneration. Skeletal muscle development includes embryonic development, postnatal growth and muscle regeneration after injury which require myogenesis upon myogenic cell proliferation, differentiation and fusion^2^. Muscle regeneration starts with the activation of muscle stem cells also termed satellite cells (SC) in response to injury^3^. During this progress, quiescent Pax7+ SC generate Pax7+/Myf5+ SC and proliferate into Pax7+/MyoD+ committed myoblasts and subsequently differentiate into Myogenin+ myocytes, which eventually fuse to repair damaged myofibers or form new multinucleated myofibers^4,5^. In addition, a portion of activated SC returns to quiescence to replenish SC pool^6,7^.

Myogenesis is highly controlled by myogenic regulatory factors (MRFs) including MyoD, Myf5, Myogenin (MyoG) and Mrf4, which can cooperate with MEF2 and bind to the E-box to induce muscle-specific genes expression^8,9^. Myogenic lineage progression is driven by Pax3, Pax7 and MRFs^10,11^, that are vital to proliferation and differentiation of muscle precursor cells. Myf5-positive SC are conducive to Pax7-dependent long-term maintenance of adult muscle stem cells^12^. Myf5-deletion myoblasts delay the proliferation and transition from proliferation to differentiation into myotubes^13,14^. In addition, Myf5 enhances myogenesis by coordinately elevating CyclinD1 transcription and translation^15^. Although both Myf5 and MyoD are essential for skeletal muscle development^16^, they have distinct functions in myogenesis. During myogenesis, Myf5 is expressed at first among myogenic transcription factors, and then MyoD is induced, while MyoG and Mrf4 are expressed later in myogenic differentiation^17–20^. MyoD as a master regulator of myogenesis converts fibroblasts into myoblasts and promotes the formation of multinucleated myotubes^21^. Knockdown of MyoG reverses terminal muscle cell differentiation^22^.

Besides MRFs, myogenesis is also tightly controlled by hierarchical interactions between transcriptional regulators, chromatin-remodeling factors and epigenetic regulation, such as DNA methylation and histone modifications, to ensure the normal proliferation and differentiation of myogenic cells^23,24^. Epigenetic modifications form a complicated network to facilitate or suppress gene expression. Of them, H3K9 and H3K27 methylation are representative transcriptional repression marks^25^.

Lysine-specific demethylase 4A (KDM4A) also known as JMJD2A is an epigenetic enzyme involved in transcriptional activation or suppression of genes through demethylation of H3K9me2/me3 and H3K36me2/me3, respectively. KDM4A belongs to KDM4 histone demethylases family implicated in multiple cellular processes including cell proliferation, cell differentiation, development and tumorigenesis^26^. Previous study indicates that KDM4B interacts with MyoD to regulate myogenic differentiation^27^. KDM4C increases MyoD transcriptional activity through inhibiting G9a-dependent MyoD degradation^28^. Recently, KDM4C has been identified as one of epigenetic regulators of DUX4-fl for targeted therapy of facioscapulohumeral muscular dystrophy^29^. KDM4D is reported to reduce H3K9me3 levels at cell cycle genes promoter in cardiac myocytes to increase heart muscle mass^30^. In addition, a new isoform of JMJD2A which lacks N-terminal domain is required for muscle differentiation^31^. It is well-known that JMJD2A promotes cardiac hypertrophy in response to hypertrophic stimuli in mice and plays a principal role in the regulation of cardiomyocytes gene^32,33^. However, the role of KDM4A in skeletal muscle development and regeneration is still unknown.

In this study, we investigated the roles of KDM4A in myogenesis and found that the conditional ablation of KDM4A in skeletal muscle caused impairment of embryonic and postnatal muscle formation. The loss of KDM4A in SC led to defective muscle regeneration and blocked the proliferation and differentiation of SC. Utilizing KDM4A gain- and loss-of-function studies, we demonstrated that KDM4A is required for activation of the key MRFs including Myf5, MyoD, MyoG by reducing H3K9me3 level on their regulatory regions to regulate myoblasts proliferation and differentiation. Altogether, our findings identify KDM4A as a key regulator of skeletal muscle development and regeneration through its roles in modulating both myogenic proliferation and differentiation.

## Materials and methods

### Mice

Myf5^Cre/+^ and KDM4A^f/f^ mice were purchased from the Jackson Laboratory (Bar Harbor, ME, USA). The KDM4A conditional KO (KDM4A cKO) strain (Control: Myf5^+/+^;KDM4A^f/f^, KDM4A cKO: Myf5^Cre/+^;KDM4A^f/f^) was generated by crossing Myf5^Cre/+^ mice and KDM4A^f/f^ mice. Mice were allocated randomly to experimental groups and processed independent on size, body weight or age. The mice used in this study had a C57BL/6J background. Housing, husbandry and all experimental protocols for mice were approved and performed in accordance with the guidelines established by the Institutional Animal Care and Use Committee of Sun Yat-sen University.

### Skeletal muscle injury

To induce muscle regeneration, 50 μL of 20 μM cardiotoxin (CTX, Millipore) was intramuscularly injected into the tibialis anterior (TA) muscle. Regenerating TA muscles were harvested at 3, 10 and 21 days post-injury.

### Satellite cells purification and culture

Satellite cells (SC) were isolated by fluorescence activated cell sorting (FACS) according to the established methods. Hindlimb muscles were minced and digested with 0.2% type II collagenase (Sigma) and 0.2% dispase (Sigma) for 1 h. The cell suspension was filtered through a 70- and 40 μm nylon filter (biosharp), and then centrifuged at 600 *g*. Washed with PBS buffer, cells were collected and stained with the following antibodies for 1 h on ice: Biotin anti-mouse/human CD11b (Biolegend, 101204), Biotin anti-mouse CD31 (Biolegend, 102404), Biotin anti-mouse CD45 (Biolegend, 103104), Biotin anti-mouse Ly−6A/E (Sca1) (Biolegend, 108103), Streptavidin-APC/Cyanine7 (Biolegend, 405208), Alexa Fluor 647 anti-mouse CD34 (Biolegend, 152205) and Anti-Integrin α7-FITC (MBL, K0046−4). Satellite cells (CD31−, CD45−, CD11b−, Sca1−, CD34 + and Integrin α7 + ) were obtained by flow cytometry analysis (Beckman MoFlo Astrios EQs). FACS-purified SCs were cultured in 24-well plates (Thermo) in Dulbecco’s Modification of Eagle’s Medium (DMEM, Corning) with 20% FBS (Gibco), basic FGF (bFGF; 10 ng/ml, Sigma) and 1% penicillin/streptomycin. Furthermore, when reaching confluence, proliferating cells were incubated with DMEM supplemented with 2% horse serum (Gibco) to induce differentiation.

### Single myofiber isolation and culture

Single myofibers were isolated from EDL muscles and digested in Dulbecco’s Modification of Eagle’s Medium (DMEM, Corning) with 0.2% NB4G collagenase (SERVA Electrophoresis, Germany) at 37 °C for 2 h. Then fibers were liberated by triturating muscle in DMEM medium with Pasteur pipettes to obtain single myofibers. Isolated single myofibers were placed in 24-well plate which had been coated with Matrigel (Corning) and cultured in DMEM with 20% fetal bovine serum, 1% penicillin/streptomycin and 10 ng/ml human basic fibroblast growth factor (bFGF, Sigma) at 37 °C with 5% CO2. After cultured for 3 or 4 days, SC migrated off the myofibers.

### Cell culture

C2C12 myoblasts were purchased from ATCC and propagated in DMEM with 10% fetal bovine serum (growth medium, GM) under moist air with 5% CO2 at 37 °C. Cells were evenly planked in 6-well plates or 24-well plates with the same cell density, three of which were randomly allocated to control or experimental groups. Post-confluent C2C12 cells were incubated with DMEM supplemented with 2% HS (differentiation medium) at the same condition for differentiation. Inducing cell differentiation was performed when cells were confluent to make sure the same cell density. Cell differentiation index (the proportion of MyHC positive cells with at least one nucleus) and fusion index (nuclei in myotubes divided by the total number of nuclei) were counted to analyze cell differentiation and fusion capacity, respectively.

### KDM4A inhibitor treatment

To inhibit KDM4A demethylase activity, cells were treated with ML324 (APExBio) 2 μM, 4 μM, 8 μM^34,35^. C2C12 cells were cultured in 24-well plates maintained in DMEM containing 10% FBS. For proliferation assay, C2C12 cells were incubated in growth medium supplemented with various concentrations of ML324 (2, 4, 8 μM) or vehicle (DMSO) for 36 h. To determine the effect of ML324 on myoblasts differentiation, confluent cells were cultured in differentiation medium supplemented with ML324 or DMSO for 2 d.

### Transfection of plasmids and siRNA

The mouse KDM4A expression plasmid was purchased from MiaoLingPlasmid (China). The coding sequence of mouse MyoD (NM_010866.2) and Myf5 (NM 008656.5) were respectively inserted into pcDNA3.1-Flag-C vector (Invitrogen, Shanghai, China). KDM4A siRNAs and negative control were purchased from Sangon Biotech (Shanghai, China). The sequences of KDM4A siRNAs were as follows: KDM4A siRNA1: 5′-GUUGAGGACAGUCUUCCCUTT-3′; KDM4A siRNA2: 5′-CAACAUUGCUGAAAGAAGUTT-3′. Transient transfections of plasmids or siRNAs were conducted with Lipofectamine 3000 (Invitrogen, USA) following manufacturer conditions. During proliferating period, fresh growth medium was exchanged to maintain cell proliferation on 12 h post-transfection. For analysis of differentiation stage, on the other hand, siRNA or plasmids were transiently transfected into confluent cells to keep the same cell density before differentiation. After transfection for 12 h, growth medium was replaced with differentiation medium to induce cell differentiation.

### Quantitative real-time PCR

RNA samples were extracted from C2C12 cells, SC or mice tissues with TRIzol reagent (Invitrogen, USA) and retro-transcribed to cDNA using the Reverse transcription kit (Promega, Shanghai, China) following manufacturer indications. Quantitative real-time PCR (qPCR) was performed in LightCycler 480 II (Roche, Basel, Switzerland) system using 2 × RealStar Green Power Mixture (Genstar, China). The expression of mRNA was normalized to expression of GAPDH. The sequence primers are listed at Supplementary Table. S1.

### EdU assay

To assay cell proliferation, C2C12 cells were incubated with EdU at a final concentration of 10 μM for 1 h in growth culture medium. After the incubation medium was removed, cells were washed in PBS and fixed with 4% PFA for 15 min. EdU staining was conducted following manufacturer’s instructions (RiboBio, China) and nucleus was stained with DAPI. Images were captured with a fluorescence microscope (Leica).

### Real-time cell proliferation monitoring assay

Real-time cell proliferation monitoring assay was conducted by RTCA xCELLigence system (ACEA biosciences, California, America). Growing cells were incubated in 16-well E-Plate for designated times and cell proliferation index was recorded by RTCA software 2.0.

### Propidium iodide (PI) flow cytometry

C2C12 cells were harvested and washed in PBS. After fixed in 70% cold ethanol at 4 °C overnight, cells were incubated in 5 mg/ml PI for 1 h at room temperature in dark. Propidium iodide (PI) flow cytometry assay was performed by the FACS Calibur Flow Cytometer (BD Biosciences, New Jersey, America).

### Western blot

Proteins were extracted from C2C12 cells or mice tissues in RIPA buffer with 1 mM PMSF (Genstar, China). Then proteins were resolved on SDS-PAGE and transferred onto 0.22 μm or 0.45 μm PVDF membranes (Millipore). Membranes were blocked using 3% BSA in TBS-Tween 0.1% for 1 h at room temperature and then incubated with primary antibodies overnight at 4 °C. After incubated with appropriate HRP-conjugated secondary antibodies, immunoblots were visualized by enhanced chemiluminescence (FDbio, China). The list of primary antibodies is shown in Supplementary Table. S2.

### Immunofluorescence

Cultured cells and muscle cross-sections were fixed in 4% PFA for 20 min and permeabilized in 0.5% Triton X−100/PBS for 15 min. Samples were then blocked in 4% BSA in PBS for 2 h at room temperature followed by incubation with primary antibodies at 4 °C overnight. Subsequently, appropriate fluorescently labeled secondary antibodies (Alexa Fluor 488, or 555) were incubated for 1 h at room temperature. DAPI was used to stain the cell nuclei for 2 min. Images were acquired with fluorescent reverse microscopy (Nikon, Japan) or confocal microscope (Leica, Germany). The antibodies used are listed in Supplementary Table. S2.

### Immunofluorescence histochemical staining analysis

Taking immunofluorescent staining for MyoG at 10 days post-injury as an example, the number of MyoG-positive cells was counted in each image. The area of fluorescent image was converted to the actual area of muscle tissue according to the length of scale. Finally, the number of MyoG-positive cells per area was calculated to compare the differentiation capacity between control and KDM4A cKO mice during muscle regeneration.

### Hematoxylin and eosin (H&E) staining

TA muscles were harvested and fixed in 4% paraformaldehyde for 48 h and subsequently embedded in paraffin. For the assessment of muscle morphology, 4 μm-thick cross-sections of TA muscles were subjected to H&E staining which was performed according to procedures provided by the H&E staining kit (Xiuwei, Guangzhou, China).

### Luciferase reporter assay

For the MyoD transcriptional activity assay, 293T cells in 24-well plate were transfected with 100 ng of the E-box-specific 4RTK-luciferase reporter (4RTK-Luc), 100 ng of MyoD expression vector, 20 ng of Renilla luciferase (RL) reporter and 100 ng of KDM4A expression vector or control vector. Cells were harvested at 48 h after transfection and luciferase activities were measured by the dual luciferase reporter assay kit (Promega).

### ChIP assay

C2C12 cells were cross‐linked in 1% formaldehyde for 10 min at room temperature and glycine was then added to a final concentration of 0.125 M for 5 min. Cells were harvested in lysis buffer (50 mM Tris-HCl pH 8.0, 10 mM EDTA, 0.5% SDS, 20 µg/ml proteinase K) on ice and chromatin was sheared using a Covaris Sonicator to obtain a fragment size distribution of 200–400 bp. Chromatin extracts were conjugated with G-protein magnetic Beads (Cell Signaling Technology) and immunoprecipitated overnight with rotation at 4 °C with 5 µg of antibodies used as follows: anti-H3K9me3, anti-KDM4A, anti-H3K4me3, anti-H3K27me3 or normal rabbit IgG, which was applied as negative control. After extensive washing, bound DNA fragments were purified and eluted by elution buffer. The enrichment of DNA sequences was analyzed and quantified by qRT-PCR using the 2 × RealStar Green Power Mixture. Primers used for ChIP-qPCR are shown in Supplementary Table. 3.

### Statistical analysis

All experiments included at least three biological replicates. Statistical analyses between different groups were performed using the Student’s *t* test or one-way or two-way ANOVA (GraphPad Software). The data are presented as the mean ± SD and the level of significance is indicated as follows: *P < 0.05, **P < 0.01, ***P < 0.001 and *P* ≥ 0.05: not significant (n.s.).

## Results

### Conditional deletion of KDM4A causes defect in muscle development

**Fig. 1:**
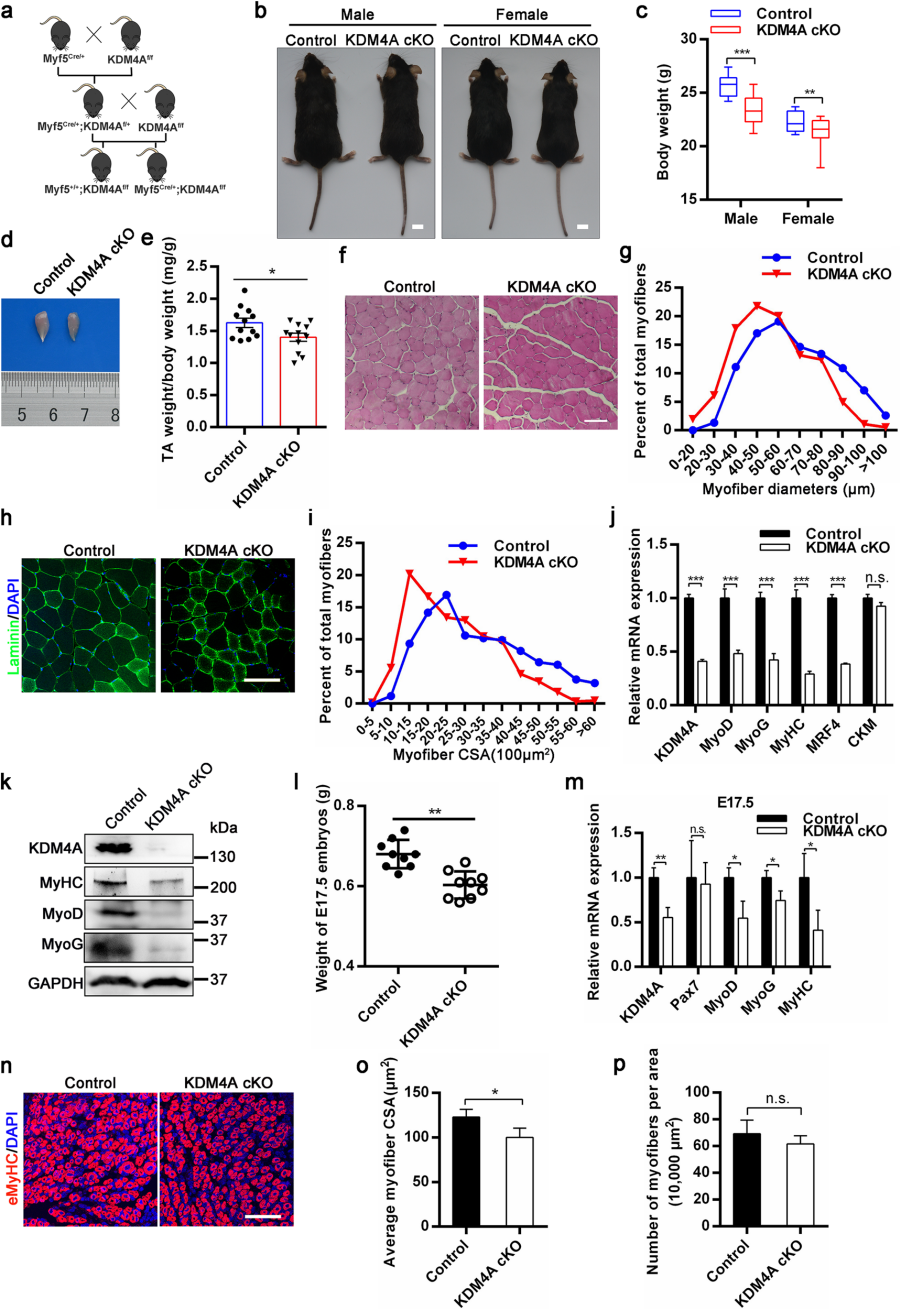
KDM4A is essential for skeletal muscle development. **a** Outline of the scheme to obtain control and KDM4A cKO mice. **b** Representative images of control and KDM4A cKO mice in 4 months. Scale bar = 1 cm. **c** The body weight of control and KDM4A cKO mice (4 months of age, *n* = 11). **d** Representative image of tibialis anterior (TA) muscle from control and KDM4A cKO mice at 4 months of age. **e** Quantification of TA weight/body weight in control and KDM4A cKO mice shown in (**d**) (*n* = 12, each). **f** Hematoxylin and eosin (H&E) staining of the TA muscle cross-sections from control and KDM4A cKO mice in 4 months. Scale bar = 100 μm. **g** Distribution of myofiber diameters of TA muscles from control and KDM4A cKO mice as described in (**f**) (*n* = 4, each). **h** Immunofluorescent staining for laminin and nuclei (DAPI) on TA muscle cross-sections of control and KDM4A cKO mice (5 months of age). Scale bar = 100 μm. **i** The distribution of fiber size measured by CSA in TA muscles from control and KDM4A cKO mice (*n* = 4, each). **j** qRT-PCR detection of the KDM4A and myogenic genes expression in the TA muscles from 4-months old control or KDM4A cKO mice (*n* = 4). **k** Protein levels of genes expression indicated in (**j**). **l** Quantifications of embryos weight from control or KDM4A cKO mice at E17.5 (*n* = 9). **m** qRT-PCR analysis showing relative transcript levels of indicated genes from E17.5 embryos. **n** Immunofluorescence analysis of eMyHC+ fibers in dorsal muscles of control and KDM4A cKO embryos at E17.5. Scale bar = 100 μm. **o**, **p** Quantifications of average myofiber CSA and the numbers of MyHC+ fibers per area in (**n**). Data are represented as mean ± SD. *P < 0.05; **P < 0.01; ***P < 0.001; n.s. not significant (Student’s *t* test).

KDM4A is ubiquitously expressed and higher in heart, skeletal muscle, and liver in mice^33^. To investigate the roles of KDM4A in skeletal muscle development, mice carrying floxed KDM4A alleles with loxP sites (KDM4Af/f) were crossed with mice expressing Cre recombinase from Myf5 locus (Myf5Cre/+) to generate conditional knock out allele, Myf5Cre/+; KDM4Af/f (thereafter referred to as KDM4A cKO), and control, Myf5 + /+; KDM4Af/f littermates (Fig. 1a). The genotype of mice was identified (Supplementary Fig. 1a). As expected, the mRNA and protein level of KDM4A were efficiently reduced in the dorsal muscle of KDM4A cKO mice (Fig. 1j, k). The body weight of KDM4A cKO mice had a significant decrease in male and female, respectively (Fig. 1b, c). Likewise, the volume and weight of TA muscles in KDM4A cKO mice were significantly lower than control (Fig. 1d, e). Hematoxylin and eosin staining and immunofluorescence staining for laminin showed that both the myofiber diameters and cross-sectional areas (CSA) of muscles in KDM4A cKO mice were significantly smaller than control mice (Fig. 1f–i). Furthermore, KDM4A cKO mice showed less mRNA and protein expression of genes related to myogenesis, such as MyoD, MyoG and MyHC (Fig. 1j, k).

To determine whether KDM4A also regulates embryonic skeletal muscle homeostasis, the expression of KDM4A from embryonic period to newborn was detected (Supplementary Fig. 1b). Moreover, the weight of E17.5 embryos and P0.5 neonatal mice were calculated (Supplementary Fig. 1c–e). Consistent with adulthood, the weight of KDM4A cKO embryos was significantly less compared to control (Fig. 1l). In addition, qRT-PCR analyses indicated the mRNA expression of myogenic regulators MyoD, MyoG, MyHC rather than Pax7 were lower in KDM4A cKO embryos (Fig. 1m). Immunofluorescence of eMyHC revealed that although there was no significance in the numbers of eMyHC+ muscle fibers, the surface area of eMyHC+ muscle fibers was significantly decreased in KDM4A cKO embryos (Fig. 1n–p). Cumulatively, these results suggest that KDM4A is essential for skeletal muscle development.

### KDM4A deletion in muscle progenitor cells impairs skeletal muscle regeneration

**Fig. 2:**
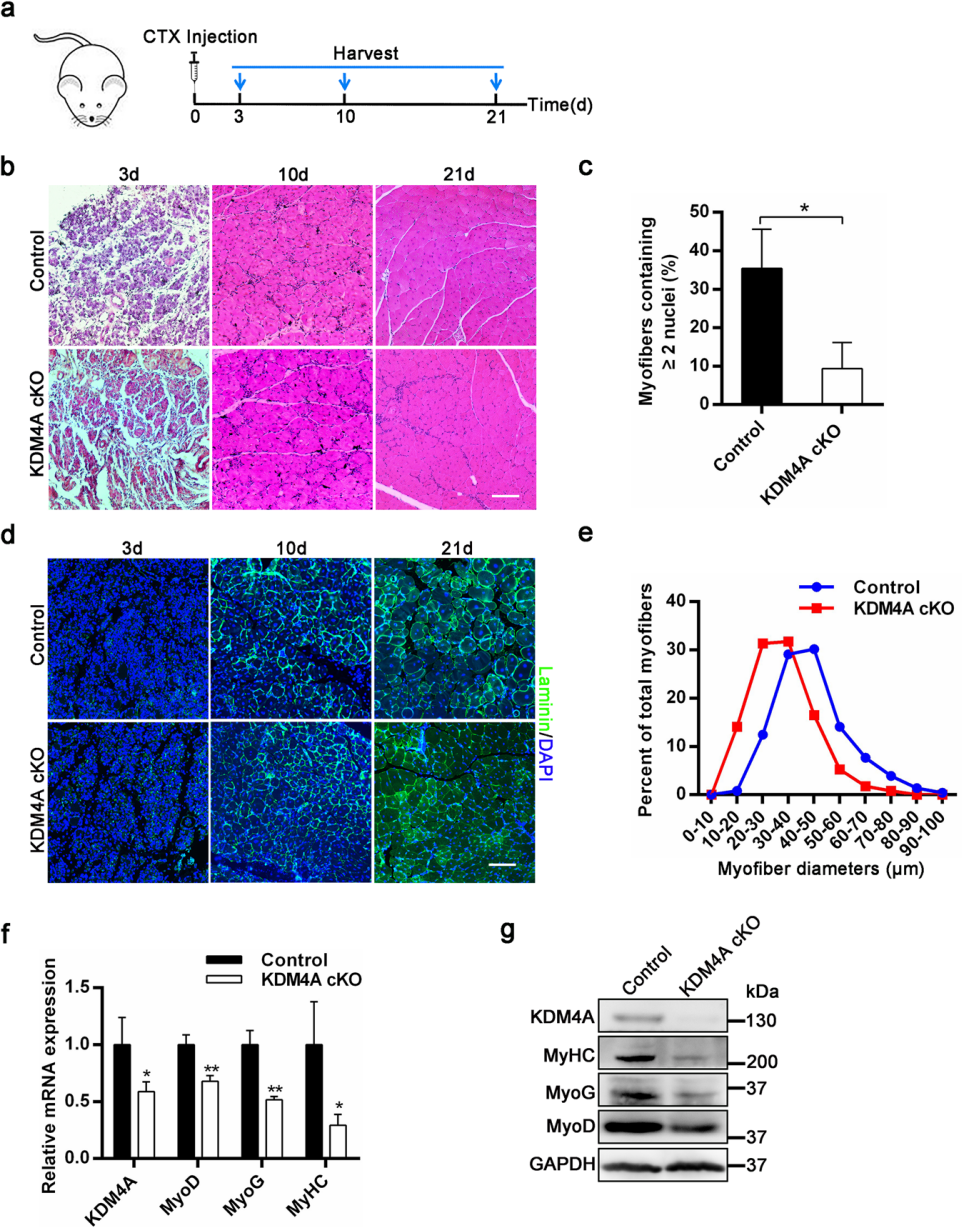
Conditional deletion of KDM4A in muscle progenitor cells impairs skeletal muscle regeneration. **a** A schematic outlining the experimental protocol followed to analyze muscle regeneration. CTX was injected into the TA muscles, then harvested and analyzed at 3 days, 10 days, 21 days post-injury. **b** Hematoxylin and eosin (H&E) staining of TA muscles from control and KDM4A cKO mice (4 months of age) at 3 days, 10 days, 21 days post-injury. Scale bar = 100 μm. **c** The percentage of myofibers containing two or more centrally located nuclei per field at 10 d post-injury (*n* = 3, each). **d** Immunofluorescence assay for laminin and nuclei on regenerating TA muscle cross-sections at at 3 days, 10 days, 21 days post-injury. Scale bar = 100 μm. **e** Distribution of myofiber diameters of CTX-injured TA muscles from control and KDM4A cKO mice at 21 days post-injury (*n* = 3). **f** qRT-PCR and **g** Western blot analysis showing the expression levels of KDM4A, MyoD, MyoG, MyHC in 10 days post-injury TA muscles from control and KDM4A cKO mice. Data are represented as mean ± SD. *P < 0.05; **P < 0.01 (Student’s *t* test).

To explore the effect of KDM4A deficiency on muscle regeneration, 4-month-old control and KDM4A cKO mice were injected with CTX into TA muscles to induce muscle injury (Fig. 2a). H&E staining on muscle cross-sections at 3, 10 and 21 days post-injury revealed a more severe regeneration defect in KDM4A cKO mice (Fig. 2b). Ten days after injury, the number of myofibers containing two or more centrally located nuclei was significantly reduced in KDM4A cKO mice (Fig. 2c). Furthermore, KDM4A cKO muscles exhibited on average significantly smaller regenerating fibers compared with control at 21 days post-injury (Fig. 2d, e and Supplementary Fig. 2a, b). In addition, mRNA expression levels of MyoD, MyoG, and MyHC were significantly lower in regenerating muscles from KDM4A cKO mice than control at 10 days post-injury (Fig. 2f); in accord with mRNA expression, protein levels of these genes were also reduced (Fig. 2g). These results demonstrate that the loss of KDM4A impairs skeletal muscle regeneration.

### KDM4A regulates the proliferation and differentiation of SCs

**Fig. 3:**
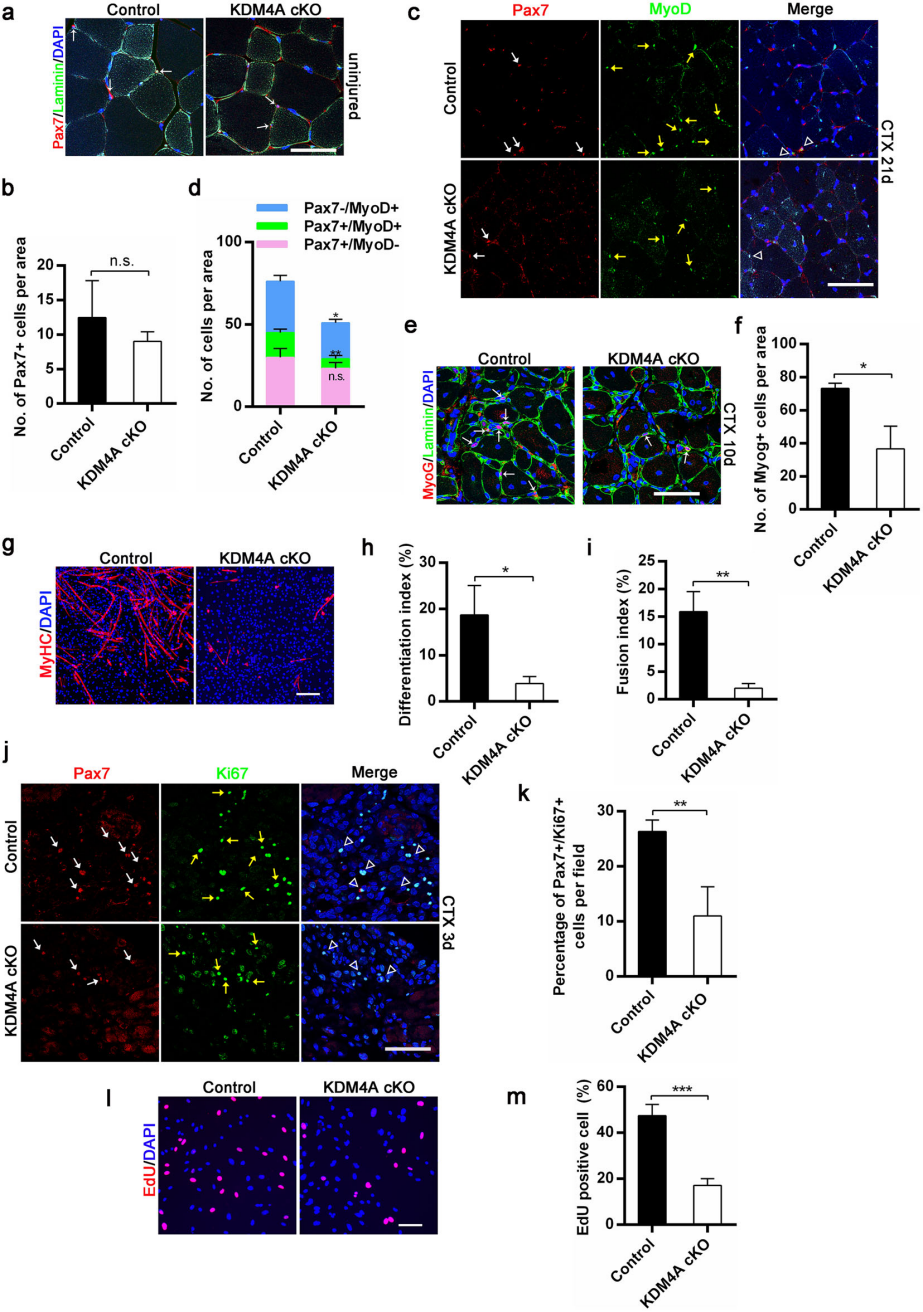
KDM4A is required for satellite cell proliferation and differentiation. **a** Immunofluorescence analysis of Pax7+ satellite cells (white arrows) in control and KDM4A cKO mice (4 months of age). Laminin staining (green) delineates the myofibers. Nuclei are counterstained with DAPI (blue). Scale bar = 50 μm. **b** Quantification of Pax7-positive cells per area of uninjured TA muscle from control and KDM4A cKO mice as shown in (**a**) (*n* = 3). **c** Immunostaining with Pax7 (red, white arrows) and MyoD (green, yellow arrows) showing a markedly reduced numbers of Pax7 + /MyoD+ cells and Pax7−/MyoD+ differentiated cells in KDM4A cKO mice at 21 days post-injury. Nuclei are counterstained with DAPI (blue). Scale bar = 50 μm. **d** Quantification of Pax7 + /MyoD−, Pax7 + /MyoD+ and Pax7−/MyoD+ cells per area from injured TA muscles of control and KDM4A cKO mice at 21 days post-injury (*n* = 3). **e** Immunofluorescence analysis of MyoG+ cells (red) in TA muscles of control and KDM4A cKO mice at 10 days post-injury. Laminin staining (green) delineates the myofibers. Nuclei are counterstained with DAPI (blue). Scale bar = 50 μm. **f** Quantification of MyoG-positive cells per area from injured TA muscle sections of control and KDM4A cKO mice at 10 days post-injury (*n* = 3). **g** Immunofluorescence staining for MyHC in FACS-sorted satellite cells from control and KDM4A cKO mice cultured for 7 d in proliferation medium followed by 3 d in differentiation medium. Scale bar = 200 μm. **h**, **i** Quantification of differentiation index (**h**) and fusion index (**i**) shown in the (**g**) (*n* = 3). **j** Immunostaining with Pax7 (red, white arrows) and Ki67 (green, yellow arrows) of TA muscles in control and KDM4A cKO mice at 3 days post-injury. Nuclei are counterstained with DAPI (blue). Scale bar = 50 μm. **k** Percentage of Pax7 + /Ki67+ double-positive cells (triangular arrowheads) per field was determined from control and KDM4A cKO TA muscles at 3 days post-injury (*n* = 3). **l** EdU staining of satellite cells isolated by FACS from control and KDM4A cKO mice cultured for 5 d in growth medium. Scale bar = 100 μm. **m** Percentage of EdU-positive cells was calculated in shown in the (**l**) (*n* = 3). Data are represented as mean ± SD. *P < 0.05; **P < 0.01; ***P < 0.001; n.s. not significant (Student’s *t* test).

Satellite cells (SC) are critical for muscle regeneration after impairment. Therefore, we investigated the importance of KDM4A to SCs. There was no significant reduction in the number of quiescent SC in control and KDM4A cKO uninjured TA muscles (Fig. 3a, b). In addition, we found no obvious change in the percentage of SCs purified by FACS between control and KDM4A cKO mice (Supplementary Fig. 3a). Consistently, the number of Pax7+ cells isolated from single myofibers did not differ between control and KDM4A cKO littermates (Supplementary Fig. 3b–d), indicating KDM4A might not be necessary for SC maintenance. Considering that the surface area of regenerating fibers was significantly decreased in KDM4A cKO mice during skeletal muscle regeneration (Fig. 2e and Supplementary Fig. 2b), we suspected that KDM4A loss might have effect on SCs differentiation. To test this notion, immunofluorescence staining for Pax7 and MyoD of injured muscles at 21 days revealed a significant reduction in Pax7 + /MyoD + (committed myoblasts) and Pax7-/MyoD+ cells (differentiated myoblasts) (Fig. 3c, d). Besides, we quantified the numbers of MyoG+ cells in regenerating TA muscle at 10 days post-injury and found a significant reduction of MyoG+ cells in KDM4A cKO mice (Fig. 3e, f). To further verify the function of KDM4A in SCs differentiation, SCs sorted by FACS were induced to differentiate followed by MyHC staining (Fig. 3g). The loss of KDM4A resulted in the lower differentiation and fusion index of SCs in KDM4A cKO mice than control (Fig. 3h, i). The above data suggested that the differentiation potential of SCs decreased when KDM4A was knocked out. Interestingly, although there was no significant difference in the number of quiescent SCs, Pax7 + /MyoD + cells at 21 days after CTX injury was reduced (Fig. 3c, d), suggesting KDM4A loss might impact the ability of SC activation or proliferation. To verify this hypothesis, immunofluorescence staining for Pax7 and Ki67 in muscle sections at 3 days post-injury (Fig. 3j), the percentage of Pax7+/Ki67+ cells per area was enumerated which revealed a dramatically reduced proliferation capacity of satellite cell in KDM4A cKO mice (Fig. 3k). Consistently, KDM4A depletion decreased SCs proliferation in vitro (Fig. 3l, m). Collectively, these results indicate that KDM4A is critical during SC proliferation and differentiation.

### KDM4A deficiency inhibits myogenic differentiation

**Fig. 4:**
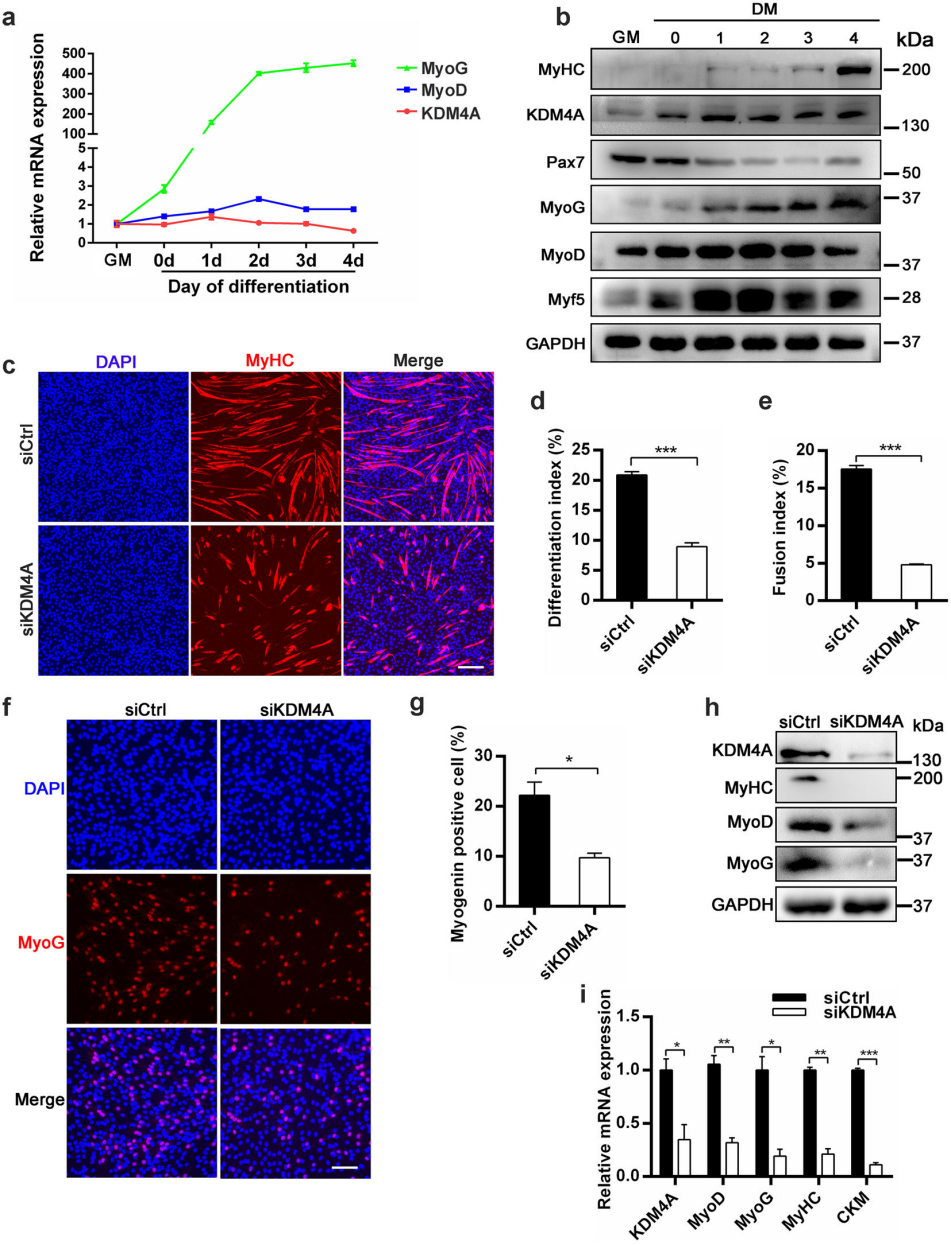
KDM4A deficiency impedes myoblasts differentiation. **a** mRNA expression profiles of KDM4A, MyoD and MyoG in C2C12 cells during differentiation. C2C12 cells were cultured in either growth medium for 2 days (GM, proliferating) or differentiation medium for 0, 1, 2, 3 or 4 d (DM 0-4). **b** Western blot analysis of C2C12 cells from GM to DM 4d was performed for the expression of KDM4A or myogenic genes and GAPDH served as loading control. **c** Immunofluorescence staining of MyHC for C2C12 cells in differentiation medium for 3 days. C2C12 cells were transfected with negative control siRNA (siCtrl) or KDM4A siRNA (siKDM4A) for 12 h in growth medium and then cultured in differentiation medium for 3 days. Scale bar = 200 μm. **d, e** Quantification of differentiation index and fusion index shown in the (**c**) (*n* = 3). **f** Representative images of immunofluorescence staining for MyoG in differentiated C2C12 cells. Cell nuclei were stained with DAPI. C2C12 cells were transfected with siCtrl or siKDM4A for 12 h and then induced to differentiate for 1 day that were stained for MyoG. Scale bar = 100 μm. **g** The number of MyoG+ cells was quantified showed that KDM4A knockdown inhibited myoblast differentiation. **h** Western blot analysis of MyoD, MyoG and MyHC protein levels in siCtrl and siKDM4A C2C12 cells after 3 d in differentiation medium. **i** mRNA expression of myogenic genes in siCtrl and siKDM4A C2C12 cells for 3 days differentiation (*n* = 3). Data are represented as mean ± SD. *P < 0.05; **P < 0.01; ***P < 0.001 (Student’s *t* test).

To study the effects of KDM4A on myogenesis in vitro, we first examined the expression profiles of KDM4A as well as MyoD and MyoG during myogenic differentiation. The protein expression level of KDM4A was increased in C2C12 cells from GM (proliferating period) to DM 1d (differentiation) similar to MyoD expression, whereas MyoG and MyHC expression gradually increased during differentiation DM 0d to DM 4d (Fig. 4a, b). Then we employed siRNA against KDM4A (siKDM4A) in C2C12 cells to silence its mRNA expression. KDM4A mRNA was effectively knocked down in siKDM4A C2C12 cells compared to control (Supplementary Fig. 4). C2C12 cells were transfected with siCtrl or siKDM4A and their differentiation was assessed by MyHC immunostaining (Fig. 4c). As a result, both myoblasts differentiation index and fusion index significantly decreased (Fig. 4d, e). The result was also strengthened by immunofluorescence staining of MyoG, which revealed the number of MyoG+ cells was diminished in KDM4A knockdown differentiated myoblasts compared to siCtrl (Fig. 4f, g). Consistently, KDM4A depletion reduced the expression of several myogenic markers, MyoD, MyoG and MyHC at both protein (Fig. 4h) and RNA (Fig. 4i) levels. Together, these data demonstrate that KDM4A deficiency impedes myogenic differentiation.

### KDM4A promotes myogenic differentiation by demethylating H3K9me3 on MyoD and MyoG regulatory regions

**Fig. 5:**
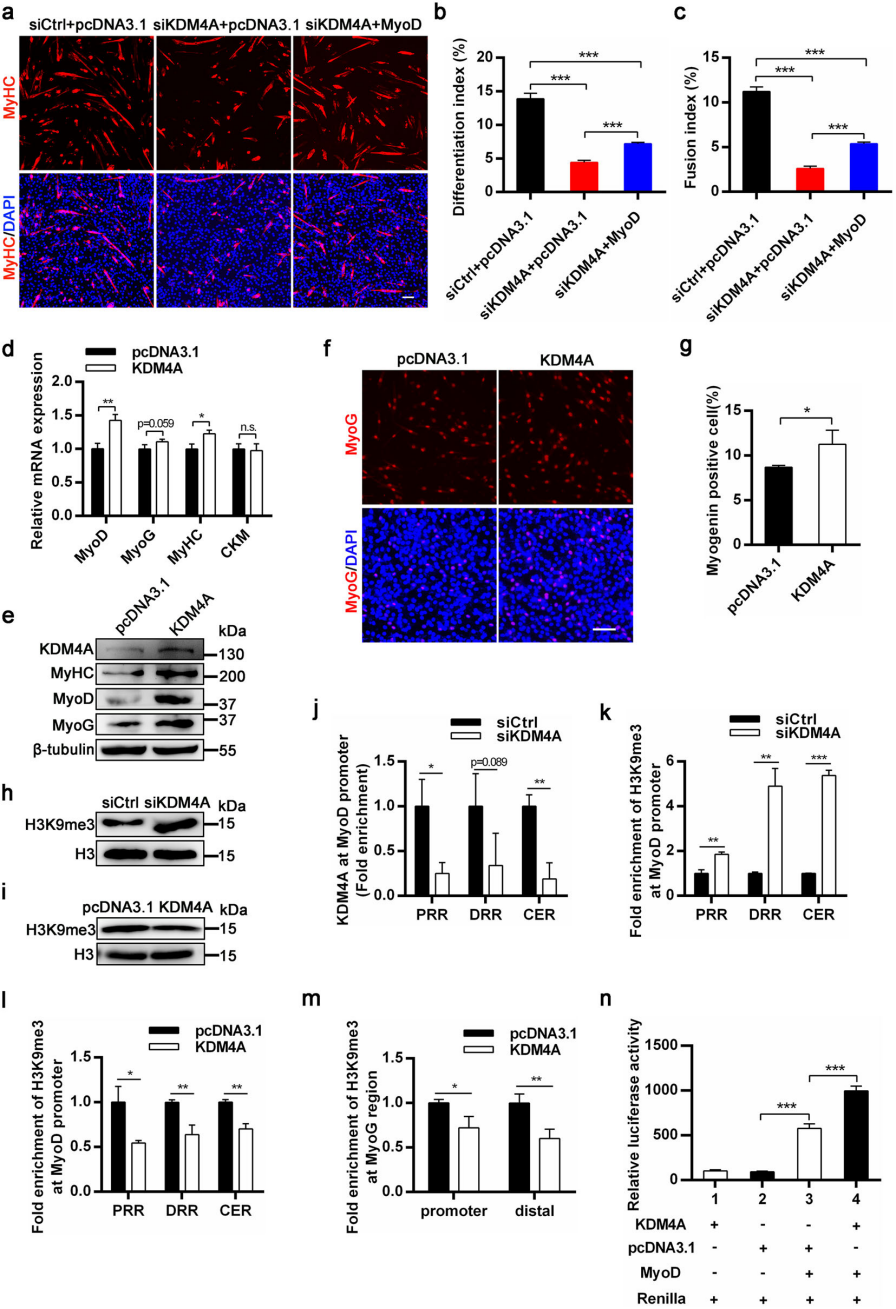
KDM4A promotes myogenic differentiation by demethylating H3K9me3 on MyoD and MyoG regulatory regions. **a** C2C12 cells were cotransfected with siCtrl or siKDM4A and pcDNA3.1 or MyoD vector. After 12 h for transfection, cells were induced to differentiate for 2 days followed by immunostaining for MyHC. Scale bar = 100 μm. **b, c** Quantification of differentiation index and fusion index represented in the (**a**) (*n* = 3). **d** Expression analysis of myogenic genes in C2C12 cells transfected with control and KDM4A plasmids at 3 d in differentiation medium using qRT-PCR. **e** Western blot analysis of KDM4A and myogenic genes protein levels in C2C12 cells transfected with control and KDM4A plasmids at 3 d in differentiation medium. **f** Immunofluorescence staining for MyoG at 1d in differentiation medium after control or KDM4A vector transfection into C2C12 cells for 12 h. Scale bar = 100 μm. **g** The number of MyoG-positive of cells was counted. **h** Western blot analysis of H3K9me3 protein levels in C2C12 cells transfected with siCtrl or siKDM4A on day 2 post-transfection with siCtrl or siKDM4A. **i** Western blot analysis of H3K9me3 protein levels in C2C12 cells on day 2 post-transfection with control or KDM4A expression vector. **j** C2C12 cells were treated with control or KDM4A siRNA for 12 h and then cultured in DM for 1 d. ChIP assay was performed to detect KDM4A enrichment at the PRR, DRR and CER region of MyoD promoter in differentiated myoblasts. **k, l** Binding of H3K9me3 to the promoters of MyoD was examined using ChIP assay on DM 1d after siCtrl or siKDM4A (**k**) and empty or KDM4A vector (**l**) transfected into C2C12 cells for 12 h (*n* = 4, each). **m** The enrichment of H3K9me3 at MyoG regulatory region in C2C12 cells stably transfected with control or KDM4A expression vector for 12 h and cultured in DM for 1 d (*n* = 4). **n** C2C12 cells were cotransfected with 4RE luciferase reporter vector and the indicated expression plasmids, then dual luciferase activities were measured after differentiation for 48 h. Data are represented as mean ± SD. *P < 0.05; **P < 0.01; ***P < 0.001 (Student’s *t* test).

We further researched the molecular mechanisms of how KDM4A regulates the myogenic differentiation. The forementioned data suggested that KDM4A Knockdown significantly inhibited myogenic differentiation along with a reductive expression of MyoD. MyoD is a master transcription factor of muscle known to orchestrate abundant muscle genes including the downregulated genes in Fig. 4, leading us to suspect that the function of KMD4A may be associated with MyoD regulation. To test this notion, control or MyoD expression vector was transfected into C2C12 cells simultaneously treated with siCtrl or siKDM4A. As anticipated, MyoD overexpression partially rescued the blunted differentiation caused by KDM4A knockdown in C2C12 cells (Fig. 5a–c). In addition, KDM4A overexpression increased the expression of myogenic marker genes MyoD, MyoG and MyHC at both protein (Fig. 5d) and RNA (Fig. 5e) levels. Meanwhile, immunofluorescence staining for differentiated myoblasts revealed a significantly elevated number of MyoG+ cells when KDM4A was overexpressed (Fig. 5f, g). These results suggested that KDM4A could promote myogenic differentiation by increasing MyoD expression (Fig. 5d–g). Previous studies have shown that KDM4A possesses a specific demethylase activity responsible for demethylation of H3K9me3, a transcriptional repressive mark. Gain- and loss-of-function revealed KDM4A knockdown augmented H3K9me3 protein levels in C2C12 cells (Fig. 5h), whereas KDM4A overexpression had the opposite effect (Fig. 5i). This result led us to explore whether KDM4A epigenetically affects the expression of MyoD gene. To address this question, we performed chromatin immunoprecipitation (ChIP) assays to determine the enrichment of H3K9me3. ChIP-qPCR assays suggested that silencing endogenous KDM4A level decreased the binding of KDM4A (Fig. 5j) and enhanced the enrichment of H3K9me3 at MyoD gene promoter (Fig. 5k), consistent with our qRT-PCR data showing a moderate decrease in MyoD mRNA level (Fig. 4i). On the contrary, KDM4A overexpression resulted in a clear and extensive decrease in H3K9me3 marks at the MyoD locus (Fig. 5l). Meanwhile, the H3K4me3 and H3K27me3 enrichment remained unchanged on all three regions (Supplementary Fig. 5a, b). Coincidentally, a modest decrease in H3K9me3 enrichment was also observed at MyoG regulatory region when KDM4A was overexpressed in C2C12 cells (Fig. 5m). To further examine the mechanisms underlying promotion of MyoD activity, we performed luciferase reporter assay using a MyoD responsive luciferase reporter 4RTK-luc, firefly luciferase reporter plasmid controlled by MyoD-binding core sequence. HEK293T cells were transiently transfected with the reporter vector and Renilla vector, together with KDM4A or MyoD plasmid (Fig. 5n). Co-transfection with KDM4A and MyoD plasmid significantly increased the transcriptional activity of 4RTK-luc compared to control. Taken together, these results indicate that KDM4A can demethylate H3K9me3 at MyoD and MyoG regulatory regions to enhance myogenic differentiation.

### KDM4A enhances skeletal muscle differentiation dependent on its demethylase activity

**Fig. 6:**
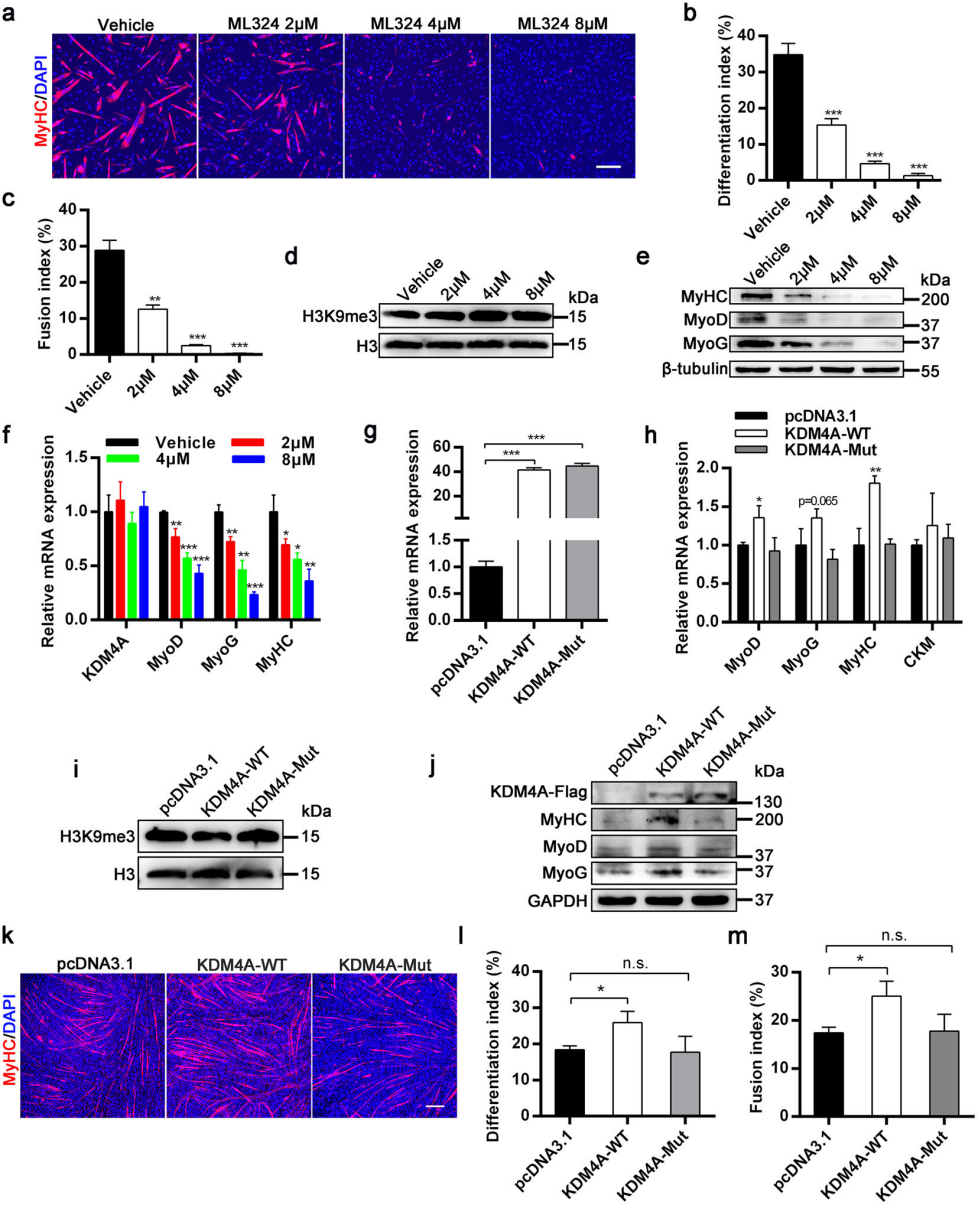
The demethylase activity of KDM4A is required for its pro-myogenic function. **a** Immunofluorescence staining for MyHC was performed to determine the effect of ML324 on myoblasts differentiation. Blue for nuclei labeled with DAPI. C2C12 cells were treated with ML324 and incubated in differentiation medium for 2 days after confluence and then stained for MyHC. Scale bar = 200 μm. **b, c** Quantification of differentiation index and fusion index as shown in (**a**). **d** Western blot analysis for H3K9me3 levels when C2C12 cells were treated with ML324 for 2 days. **e** Protein levels of MyoD, MyoG and MyHC in C2C12 cells treated with vehicle or ML324 and cultured in DM for 2 d. **f** mRNA levels of KDM4A and myogenic factors were validated by qPCR as described in the (**e**). **g** Overexpression of KDM4A in C2C12 cells was verified using qRT-PCR. **h** C2C12 cells were transfected with empty, KDM4A-WT and KDM4A-Mut plasmid for 12 h and then induced to differentiate for 2 d. The expression of the myogenic marker genes was analyzed by qRT-PCR. **i** The protein levels of H3K9me3 were checked after empty, KDM4A-WT and KDM4A-Mut plasmid transfected into C2C12 cells for 2 d in differentiation medium. **j** KDM4A-Flag and muscle-specific genes protein levels in C2C12 myotubes transfected with empty, KDM4A-WT and KDM4A-Mut plasmid were analyzed by Western blot analysis. **k** C2C12 myoblasts stably transfected with empty, KDM4A-WT or KDM4A-Mut plasmid were cultured in DM for 3 days. Cells then were immunofluorescence stained for MyHC. Scale bar = 200 μm. **l, m** Quantification of differentiation index and fusion index represented in the (**k**) (*n* = 3). Data are represented as mean ± SD. *P < 0.05; **P < 0.01; ***P < 0.001; n.s. not significant (Student’s *t* test).

To address the significance of demethylase activity on myogenesis, we employed ML324 that has been reported to be an effective inhibitor of KDM4A activity^34^ to treat C2C12 cells. As expected, the H3K9me3 protein levels were dramatically increased in C2C12 cells with ML324 treatment (Fig. 6d). Subsequently, we measured the effects of ML324 on differentiation by immunofluorescence experiments (Fig. 6a) and found ML324 repressed myoblasts differentiation and fusion in a dose-dependent manner (Fig. 6b, c). Especially when treated with 8 μM ML324, C2C12 cells scarcely formed multinucleated myotubes. Furthermore, biochemical analysis through western blot (Fig. 6e) and qRT-PCR (Fig. 6f) showed remarkably decreased expression of muscle markers in a dose-dependent manner.

To further evaluate the requirement of KDM4A demethylase activity on myoblasts differentiation in vitro, we constructed demethylase-deficient mutant of KDM4A (KDM4A^H188A^). qRT-PCR results demonstrated that the expression levels of KDM4A were efficiently increased in C2C12 cells after transfection with either wildtype (KDM4A-WT) or mutant KDM4A construct (KDM4A-Mut) (Fig. 6g). Expectedly, overexpression of KDM4A-WT in C2C12 cells significantly reduced the level of H3K9me3, while the mutant KDM4A did not (Fig. 6i). Moreover, the mRNA and protein expression of myogenic genes were increased in C2C12 cells overexpressing KDM4A-WT rather than KDM4A-Mut compared with control vector (Fig. 6h, j). Accordingly, overexpression of KDM4A-WT in C2C12 cells accelerated myoblasts differentiation. By contrast, the KDM4A-Mut had no pro-myogenic effect (Fig. 6k–m). Altogether, the above findings indicate that KDM4A enhances myogenic differentiation dependent on its demethylase activity.

### KDM4A accelerates myoblasts proliferation via decreasing H3K9me3 enrichment at Myf5 promoter

**Fig. 7:**
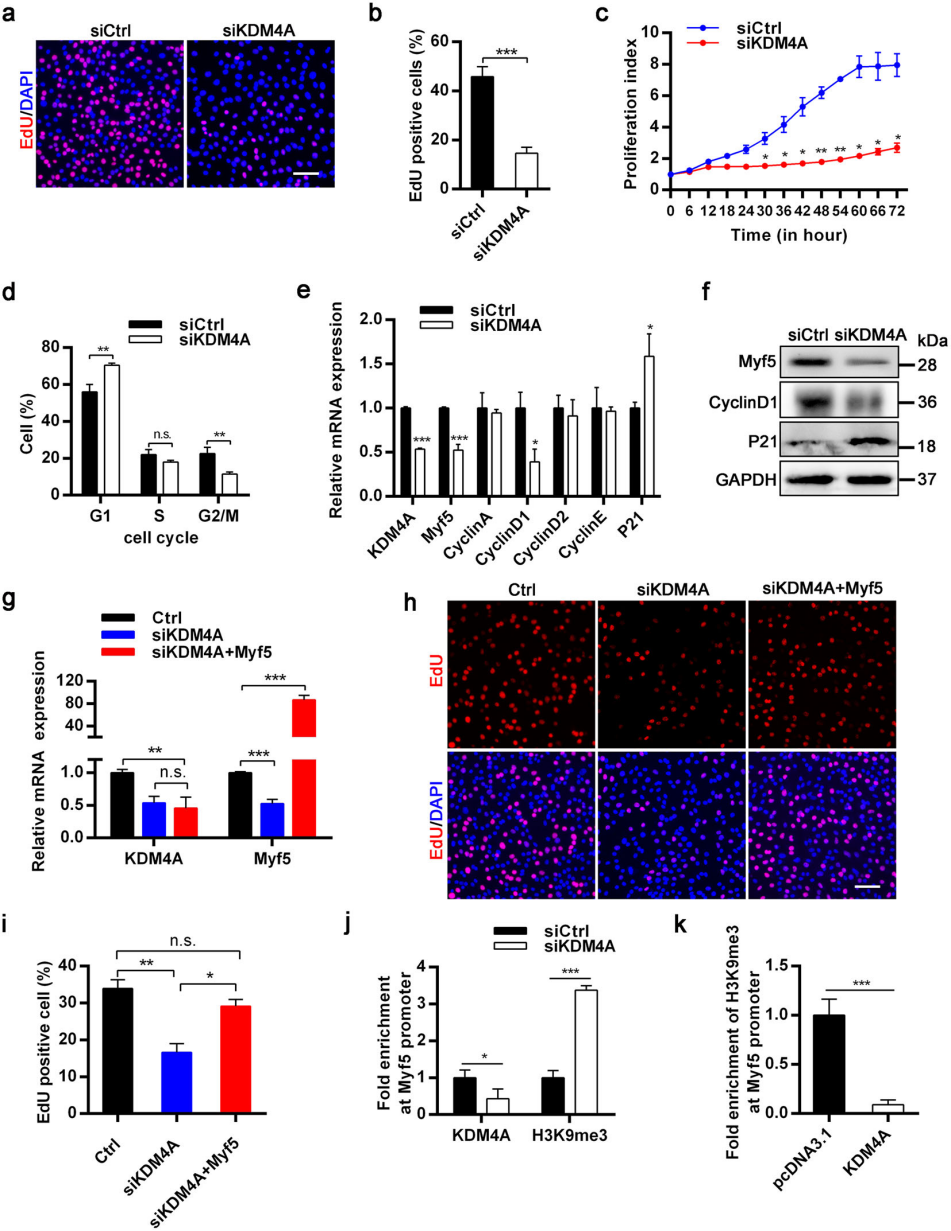
KDM4A facilitates myoblasts proliferation by demethylating H3K9me3 at Myf5 loci. **a** Representative images of the EdU staining for siCtrl or siKDM4A C2C12 cells on 36 h post-transfection. Scale bar = 100 μm. **b** Quantification of the percentage of EdU-positive cells in (**a**). **c** Real-time cell proliferation monitoring assay of the proliferation index of C2C12 cells. **d** The cell cycle distribution of proliferating myoblasts was analyzed through flow cytometry after PI staining. C2C12 cells were transfected with siCtrl and siKDM4A for 36 h. **e** Expression analysis of cell-cycle related genes in siCtrl and siKDM4A C2C12 cells in growth medium using qRT-PCR. **f** Western blot showing Myf5, Cyclin D1, P21 and GAPDH levels in the same conditions as in (**e**). **g** qRT-PCR showing the mRNA expression levels of KDM4A and Myf5 in proliferating C2C12 cells transfected with siRNA or plasmids as indicated. **h** EdU staining of C2C12 cells cotransfected with siCtrl or siKDM4A and control or Myf5 plasmid for 36 h in growth medium. **i** The number of EdU-positive cells was counted. **j** ChIP-qPCR analysis of the binding of KDM4A and H3K9me3 at My5 promoter in siCtrl and siKDM4A C2C12 cells. **k** The enrichment of H3K9me3 at Myf5 locus in C2C12 myoblasts transduced with either empty plasmid or KDM4A plasmid. Data are represented as mean ± SD. *P < 0.05; **P < 0.01; ***P < 0.001; n.s. not significant (Student’s *t* test).

Our abovementioned results showed that KDM4A is required for SCs proliferation (Fig. 3j–m). As a consequence, we further investigated if and how KDM4A regulates myoblasts proliferation during myogenesis. EdU staining assay indicated KDM4A knockdown inhibited myoblasts proliferation (Fig. 7a, b), that was also confirmed by immunofluorescent staining for Ki67 (Supplementary Fig. 6a, b). Moreover, the proliferation index of myoblasts was remarkably declined when KDM4A was silenced in C2C12 cells using real-time cell proliferation monitoring assay (Fig. 7c). By contrast, overexpression of KDM4A boosted the proliferation potential of myoblasts (Supplementary Fig. 6c). In addition, knockdown of KDM4A prominently increased the population of cells in G1 phase whereas G2/M populations strikingly diminished, revealing an arrest of cell cycle in G1 phase (Fig. 7d). Silencing KDM4A in C2C12 cells markedly reduced the expression of Cyclin D1 (cell cycle regulators involved in G1/S transition) and elevated the expression of P21 (cyclin‐dependent kinase inhibitor 1 A) (Fig. 7e, f), while KDM4A overexpression had the opposite effects (Supplementary Fig. 6e). Interestingly, we also observed KDM4A depletion hampered Myf5 expression of both mRNA and protein levels (Fig. 7e, f), while KDM4A overexpression accelerated the mRNA expression of Myf5 (Supplementary Fig. 6d). Previous studies have shown that Myf5 enhances translation and modestly increases transcription of Cyclin D1 to promote myogenesis^15^. Therefore, we hypothesized that KDM4A regulates myogenesis in part by Myf5 allowing for the aforementioned data. To examine this speculation, C2C12 myoblasts stably expressing control vector or Myf5 vector were transfected with siCtrl or siKDM4A in growth media (Fig. 7g). Myf5 overexpression rescued the attenuated proliferation of C2C12 myoblasts (Fig. 7h, i). To dissect whether KDM4A epigenetically regulates the expression of Myf5, we performed ChIP-qPCR assay using an anti-H3K9me3 antibody in siCtrl or siKDM4A C2C12 myoblasts and analyzed H3K9me3 enrichment at Myf5 promoter. KDM4A depletion contributed to a clear increase in H3K9me3 marks at the Myf5 locus (Fig. 7j). Rather, reduced occupancy of H3K9me3 at Myf5 promoter was observed in KDM4A-overexpressing myoblasts (Fig. 7k), though the binding of H3K4me3 and H3K27me3 remained unchanged (Supplementary Fig. 6f). To further evaluate the demethylation role of KDM4A on Myf5 expression, C2C12 cells were treated with either vehicle or ML324. Myf5 and Cyclin D1 levels were dramatically decreased, while P21 protein level was promoted in the presence of ML324 (Supplementary Fig. 6g). In summary, these data clearly indicate that KDM4A demethylates H3K9me3 at Myf5 promoter to increase Myf5 expression which subsequently enhances the expression of Cyclin D1.

## Discussion

Skeletal muscle relies on myogenic cells to maintain homeostasis. Increasing studies have revealed that epigenetic factors are pivotal for muscle cell lineage commitment, proliferation, differentiation and formation to myofibers^36^. Recent reports show that KDM4A is ubiquitously expressed and higher in the heart, skeletal muscle, and liver^33^, which promotes cardiac hypertrophy in response to hypertrophic stimuli in mice. Similarly, another finding reveals high expression levels of KDM4A in bone and skeletal muscle in mice, regulating adipogenic and osteogenic differentiation via epigenetic regulation of C/EBPα and canonical Wnt signaling^35^. However, the role of KDM4A in myogenesis and skeletal muscle development is still unknown. In this study, we demonstrated that skeletal muscle development was impaired in either embryonic or adult KDM4A cKO mice by decreasing the expression of myogenic genes. Furthermore, knockdown of KDM4A dramatically lesioned muscle regeneration in vivo. Compared with control, KDM4A cKO mice has smaller repaired myofibers and more serious inflammatory response, resulting in the delayed muscle regeneration. This result was proved by the attenuated muscle-specific genes expression. Taken together, our study underscored the key functions of KDM4A in regulating muscle development and regeneration.

Satellite cells (SC) are indispensable for skeletal muscle regeneration^5^. In the current study, we found KDM4A participated in regulating SC fate. During skeletal muscle regeneration, KDM4A-deficient SC failed to differentiate, as evidence by the reduction of MyoG in vivo and MyHC for FACS-sorted SCs in vitro experiments. It is widely known that MyoG is a key factor to SC differentiation and myocytes fusion^3,37^. Especially, skeletal muscle does not develop normally in the absence of MyoG^38^. Muscle development relies on proliferation and differentiation potential of SCs. We found loss of KDM4A had no obvious effect on the number of quiescent SCs. However, reduced Pax7+/MyoD+ expressing cells were observed at 21 days post-injury indicating it appeared to be largely due to a defect in satellite cell activation or proliferation. Furthermore, Pax7+/Ki67+ cells at 3 days post-injury and EdU+ FACS-purified SCs were decreased suggesting KDM4A has a high potential to impact the proliferation ability of SC. Our findings confirm that KDM4A is a critical factor for muscle stem cells function and may suggest that KDM4A plays important roles in other stem cell types. Previous studies show that KDM4A is required for hematopoietic stem cell maintenance depending on its histone demethylase^39^. Inhibiting KDM4A blocks breast cancer stem-like cells proliferation used as therapy for resistant cancer stem-like cells^40^. In addition, KDM4A regulates embryonic stem cells (ESC) self-renewal and differentiation to endothelial cells which is essential for early embryonic development^41,42^. As a novel epigenetic regulator, KDM4A promotes adipogenic differentiation and blocks osteogenic differentiation of marrow stromal progenitor cells^35^.

During muscle regeneration, quiescent SCs are activated and committed to myoblasts and subsequently differentiate into myocytes, which eventually fuse to repair damaged myofibers or form new multinucleated myofibers^4,43^. In this study, we found KDM4A was essential for myoblasts differentiation and fusion dependent on its demethylase. However, previous finding has reported that an isoform of histone demethylase JMJD2A/KDM4A lacking the N-terminal demethylase domain is necessary for myotube formation^31^. These indicate KDM4A may regulate myogenic differentiation through different ways. We found deletion of KDM4A resulted in downregulation of genes including MyoD and MyoG in vitro and in vivo experiments, which are master transcription factors of myogenesis and belong to the MRFs. MRFs regulate cell fate determination and terminal differentiation of the myogenic precursors in a multistep process that eventually culminate with formation of muscle fibers^9^.

Recent evidences have revealed that changes in histone methylation modifications of key transcription factors are responsible and crucial for muscle differentiation^26,44^. Ezh2-mediated methylation of H3-K27 suppresses the expression of MyoG and MyHC, resulting in decreased myogenesis^45^. G9a impedes myoblasts differentiation by mediating H3K9me2 on MyoD target promoters^25^. Jmjd2C increases myogenic conversion and MyoD transcriptional activity with erasing repressive H3K9me3 level at the promoter of MyoD target genes^28^. In the present study, we found the enrichment of H3K9me3 was significantly increased on MyoD regulatory sequences in the absence of KDM4A. In contrast, overexpression of KDM4A led to the lessened occupancy of H3K9me3 at MyoD locus. It is widely spread that MyoG is a key factor required for SC differentiation and myocyte fusion tightly controlled by MyoD. Likewise, KDM4A eliminated H3K9me3 level at MyoG loci in accordance with previous data^31^. Moreover, KDM4A was conducive to MyoD transcriptional activities. Nevertheless, KDM4A lost the pro-myogenic differentiation capacity when its demethylase activity was restrained. Mechanistically, these results suggest KDM4A regulates the expression of muscle-specific genes such as MyoD and MyoG through removing repressive mark H3K9me3 level on their regulatory regions.

The proliferation and differentiation of muscle precursor cells demand the coordinated activity of MRFs including Myf5. Previous studies have shown that Myf5 enhances Cyclin D1 translation and transcription to promote myogenesis^15^. Given these evidences, our findings indicate that KDM4A maintains myoblasts proliferation by regulating the expression of Myf5 and Cyclin D1. The decreased proliferation potential due to lack of KDM4A was rescued by Myf5 overexpression. With the lower occupancy of KDM4A, the enrichment of H3K9me3 mark at Myf5 promoter was dramatically augmented. Meanwhile, attenuated Myf5 level subsequently declined Cyclin D1 expression accompanied by the enhanced expression of P21, resulting in defective proliferation and arrest of cell cycle in G1 phase. Similarly, a previous finding also has shown that the inhibition of KDM4A by IOX1 suppresses cell proliferation, migration and cell cycle progression of vascular smooth muscle cells by inhibiting Cyclin D1 expression and increasing P21 expression^46^. Further studies find that SC from Myf5 KO mice have proliferation defects^13^. Collectively, KDM4A may promote myogenic program via regulating the expression of Myf5. In the proliferating myoblasts, MyoD gene is transcribed in a moderate level through a TFIID-dependent mechanism and spatial localization near the periphery of the nucleus^47^. However, although MyoD can be recruited to the binding sites of MyoG gene, it still does not initiate the transcription of MyoG at the stage of proliferation due to complicated networks including repressive epigenetic modifications, as the evidence that stable expression of MyoG in primary myoblasts has never been reported. When myoblasts differentiate toward myotubes, MyoD gene moves toward the lumen of the nucleus by a TAF3/TRF3-dependent transcriptional mechanism^48^ and up-regulated expression of KDM4A enhances the transcription of MyoD, resulting in a higher level of MyoD expression. The transcriptional activation of MyoG requires an open chromatin structure established by MyoD^49^. Under these conditions, KDM4A removes the H3K9me3 on MyoG region and can serve as transcriptional coactivator with MyoD to elevate MyoG transcription, thus accelerating myogenic differentiation. Further endeavors will be devoted to determining the detailed mechanisms by which KDM4A affects the switch of proliferation and differentiation to regulate myogenic program.

**Fig. 8:**
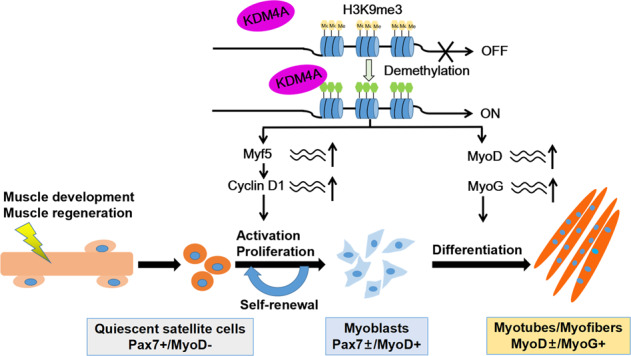
Schematic model of KDM4A regulation during myogenesis. In the proliferating myoblasts, KDM4A demethylates H3K9me3 at Myf5 promoter. Subsequently, upregulation of Myf5 enhances Cyclin D1 expression, leading to the promotion of myogenic cell proliferation. Upon differentiation, KDM4A decreases the enrichment of H3K9me3 at both MyoD and MyoG loci to promote the expression of muscle-specific genes including MyoD, MyoG and MyHC, thus facilitating myogenic differentiation.

In conclusion, our results implicate KDM4A is a positive regulator of myogenesis that is essential for skeletal muscle development and regeneration. KDM4A promotes myogenic differentiation through removing H3K9me3 modification on MyoD and MyoG regulatory regions. On the other hand, KDM4A maintains myoblasts proliferation by enhancing Myf5 expression in a demethylase activity-dependent manner and subsequently elevating Cyclin D1 level (Fig. 8). Our findings may provide new insight for future therapies aimed at treating myogenic deficiencies or malignant diseases.

## Supplementary information

Supplementary Figure legends

Supplementary tables

Supplementary Figure 1

Supplementary Figure 2

Supplementary Figure 3

Supplementary Figure 4

Supplementary Figure 5

Supplementary Figure 6
