# Supplementary material for: KDM4A regulates myogenesis by demethylating H3K9me3 of myogenic regulatory factors
Source: Cell Death Dis. 2021 May 19;12(6):514. doi: 10.1038/s41419-021-03799-1 (PMC8134519; doi:10.1038/s41419-021-03799-1)
Supplement: Supplementary file 1 — Supplementary Figure legends [file 41419_2021_3799_MOESM1_ESM.pdf]

KDM4A regulates skeletal muscle development and regeneration

Qi Zhu, Feng Liang, Shufang Cai, Xiaorong Luo, Tianqi Duo, Ziyun Liang, Zuyong He, Yaosheng Chen, Delin Mo*

Fig. S1 KDM4A affects embryonic muscle development. a Agarose gel electrophoresis detected the genotype of mice. **b** qRT-PCR analysis for the expression of KDM4A and MyoD in the whole embryo (E9) and dorsal muscle (E11-P4) at the indicated ages (n = 3). **c d** Representative images of control and KDM4A cKO embryo at E17.5 and newborn mice at P0.5, respectively. Scale bar = 1 cm. **e** The body weight of P0.5 neonatal mice (n = 10, left; n = 7, right). Data are represented as mean ± SD. *$P<0.05$ (Student's t-test).

Fig. S2 KDM4A knockout delays muscle regeneration. a Immunofluorescence assay for MyHC and nuclei on regenerating TA muscle cross sections at 10 days, 21 days post-injury. Scale bar = 100 μm. **b** CSA measurement of regenerating muscle myofibers from control and KDM4A cKO mice at 21 days after CTX treatment (n = 3).

Fig. S3 Isolation and identification of satellite cells. a Representative FACS plots showing the percentage of SCs sorted from 4-month-old control and KDM4A cKO mice (n = 3, each). **b** Representative image of isolated live single myofiber. Satellite cells migrated from the myofiber when cultured for 3d. Scale bar = 200 μm. **c** Myofiber was removed and the remained cells were stained for Pax7. Nuclei are counterstained with DAPI. Scale bar = 100 μm. **d** Percentage of Pax7 positive cells in panel c was calculated. Data are represented as mean ± SD. n.s., not significant (Student's t-test).

Fig. S4 Knockdown efficiency determination of siRNAs against KDM4A. C2C12 cells were transfected with siCtrl, siKDM4A-1 or siKDM4A-2 for 36 h. The efficiency of siRNAs to silence KDM4A in C2C12 cells was determined by qRT-PCR. Data are represented as mean ± SD. ***$P<0.001$ (Student's t-test).

Fig. S5 KDM4A has no obvious effect on the enrichment of H3K4me3 and H3K27me3 at MyoD loci. a b ChIP-qPCR dissected the occupancy of H3K4me3 and H3K27me3 on different MyoD regulatory regions when KDM4A was overexpressed in C2C12 cells, respectively. Data are presented as mean ± SD. n.s., not significant (Student's t-test).

Fig. S6 KDM4A promotes myoblasts proliferation dependent on demethylase cativity. a Immunofluorescence staining for Ki67 was performed to determine the proliferation of siCtrl or siKDM4A C2C12 myoblasts on 48 h post-transfection. Scale

bar = 100 μm. **b** Quantification of Ki67 positive cells as shown in a. **c** Real-time cell proliferation monitoring assay analysis the proliferation index of C2C12 cells transfected with empty or KDM4A plasmid. **d** qRT-PCR showing the mRNA expression levels of KDM4A and Myf5 in proliferating C2C12 cells transfected with control or KDM4A vector for 36 h. **e** Expression analysis of cell-cycle related genes in control or KDM4A-overexpression C2C12 cells using qRT-PCR. **f** The enrichment of H3K4me3 and H3K27me3 at Myf5 promoter in C2C12 cells stably transfected with control or KDM4A expression vector was examined. **g** Western blot analysis of Myf5, Cyclin D1 and P21 protein levels in C2C12 cells treated with vehicle or ML324 and incubated in growth medium for 36 h. Data are represented as mean ± SD. n = 3 per group. *$P<0.05$; **$P<0.01$; ***$P<0.001$; n.s., not significance (Student's t-test).
