# Supplementary material for: KDM4A regulates myogenesis by demethylating H3K9me3 of myogenic regulatory factors
Source: Cell Death Dis. 2021 May 19;12(6):514. doi: 10.1038/s41419-021-03799-1 (PMC8134519; doi:10.1038/s41419-021-03799-1)
Supplement: Supplementary file 2 — Supplementary tables [file 41419_2021_3799_MOESM2_ESM.pdf]

Table S1 The primers for qRT-PCR

Gene	Sequence	
GAPDH	F:AGGTCGGTGTGAACGGATTTG	R:TGTAGACCATGTAGTTGAGGTCA
MyoD	F:CCACTCCGGGACATAGACTTG	R:AAAAGCGCAGGTCTGGTGAG
MyoG	F:GAGACATCCCCCTATTTCTACCA	R:GCTCAGTCCGCTCATAGCC
KDM4A	F:GAGGAAGACTGCTGCTTATGCTC	R:TCACATCCACTGGACTTCTTTCA
Pax7	F:GAATCAGAACCCGACCTCCC	R:CGCCGGTTACTGAACCAGA
Myf5	F:CCTGTCTGGTCCCGAAAGAAC	R:GACGTGATCCGATCCACAATG
MyHC	F:AAAAGGCCATCACTGACGC	R:CAGCTCTCTGATCCGTGTCTC
CKM	F:CTGACCCCTGACCTCTACAAT	R:CATGGCGGTCCTGGATGAT
P21	F:CCTGGTGATGTCCGACCTG	R:CCATGAGCGCATCGCAATC
Cyclin A	F:AAGAGAATGTCAACCCCGAAAAA	R: ACCCGTCGAGTCTTGAGCTT
Cyclin D1	F:GCGTACCCTGACACCAATCTC	R:CTCCTCTTCGCACTTCTGCTC
Cyclin D2	F:GAGTGGGAACTGGTAGTGTTG	R:CGCACAGAGCGATGAAGGT
Cyclin E	F:ATGTCAAGACGCAGCCGTTTA	R:GCTGATTCCTCCAGACAGTACA
MRF4	F:CTACATTGAGCGTCTACAGGACC	R:CTGAAGACTGCTGGAGGCTG

Table S2 Antibodies and their application

Antibody name	Catalogue Number	Brand name	Dilution ratio		
			WB	IF	ChIP
Anti-rabbit IgG, HRP-linked Antibody	7074S	Cell Signaling	1:1000		
Anti-mouse IgG, HRP-linked Antibody	7076S	Cell Signaling	1:1000		
Anti-rabbit IgG(H+L),F(ab')2 Fragment (Alexa Fluor 488 Conjugate)	4412S	Cell Signaling		1:1000	
Anti-rabbit IgG(H+L),F(ab')2 Fragment (Alexa Fluor 555 Conjugate)	4413S	Cell Signaling		1:1000	
Anti-mouse IgG (H+L),F(ab')2 Fragment (Alexa Fluor 555 Conjugate)	4409S	Cell Signaling		1:1000	
anti-GAPDH	AP0063	Bioworld		1:5000	
anti-KDM4A	A300-861A	Bethyl	1:1000		
anti-JMJD2A	ab105953	abcam			1：50
anti-MyoD	ab16148	abcam	1:1000		
anti-Myogenin	ab1835	abcam	1:1000	1:500	
anti-eMyHC	BF-G6	DSHB		1:100	
anti-MyHC	ab51263	abcam	1:1000	1:500	
anti-Myf5	ab125078	abcam	1:1000		

anti-Cyclin D1	2978T	Cell Signaling	1:1000		
anti-P21	ab188224	abcam	1:1000		
anti-β-Tubulin	2148S	Cell Signaling	1:1000		
anti-Laminin	L9393	Sigma		1:200	
anti-H3K9me3	ab8898	abcam	1:1000		1:50
anti-Pax7	MF20	DHSB		1:200	
anti-Ki67	ab15580	abcam		1:500	
anti-H3	ab201456	abcam	1:1000		
anti-IgG	2729S	Cell Signaling			1:200
anti-Flag-tag	#250111	Zen BioScience	1:1000		

Table S3 The primers for ChIP-qPCR

Gene	Sequence	
MyoD PRR	F:GGACCCCAAGCTCCGCCCTACTACA	R:TAAAAGCCCGGGAGCTGGAAAGG
MyoD DRR	F:AGTCCTTCAGCCCCCTAGACCCAAG	R:AACTAGCACCTGCCCCAAGCCTCAG
MyoD CER	F:GCTTCTTTCGGCCAAGTATCCTCCTCC	R:CTGGCTGTGTTGTGAGTCACGGGTT
MyoG promoter	F:GAAGGGGAATCACATGTAATCCACTGGA	R:ACGCCAACTGCTGGGTGCCATT
MyoG distal	F:CGGGGTACCATTCTAGAGTTGTATGACGCAGGC	R:GGTCGATAAGGAGAAAGAG
Myf5 promoter	F:TCCAGAAGGCCACCGAG	R:TTATTAGCATATCCCACCACAAC

Table S4 The primers for genotype of Myf5$^{Cre/+}$; KDM4A$^{f/f}$ mice

No.	Name	Sequence	Product length (bp)
1	Myf5-common-F	AACCAGAGACTCCCCAAGGT	WT: 240
	Myf5-WT-R	CGGCTCTTAAAGCAATGGTC	
2	Myf5-common-F	AACCAGAGACTCCCCAAGGT	Cre: 120
	Myf-mutant-R	ACGAAGTTATTAGGTCCCTCGAC	
3	KDM4A-WT-F	CCTCGAACATCCTACGACGA	WT: 300
	KDM4A-common-R	GGAGGGCGATGTATTTCTACTG	
4	KDM4A-mutant-F	GTGGGCTCTATGGCTTCTGA	floxp: 240
	KDM4A-common-R	GGAGGGCGATGTATTTCTACTG	
