# Supplementary figures and images for: KDM4A regulates myogenesis by demethylating H3K9me3 of myogenic regulatory factors
Source: Cell Death Dis. 2021 May 19;12(6):514. doi: 10.1038/s41419-021-03799-1 (PMC8134519; doi:10.1038/s41419-021-03799-1)

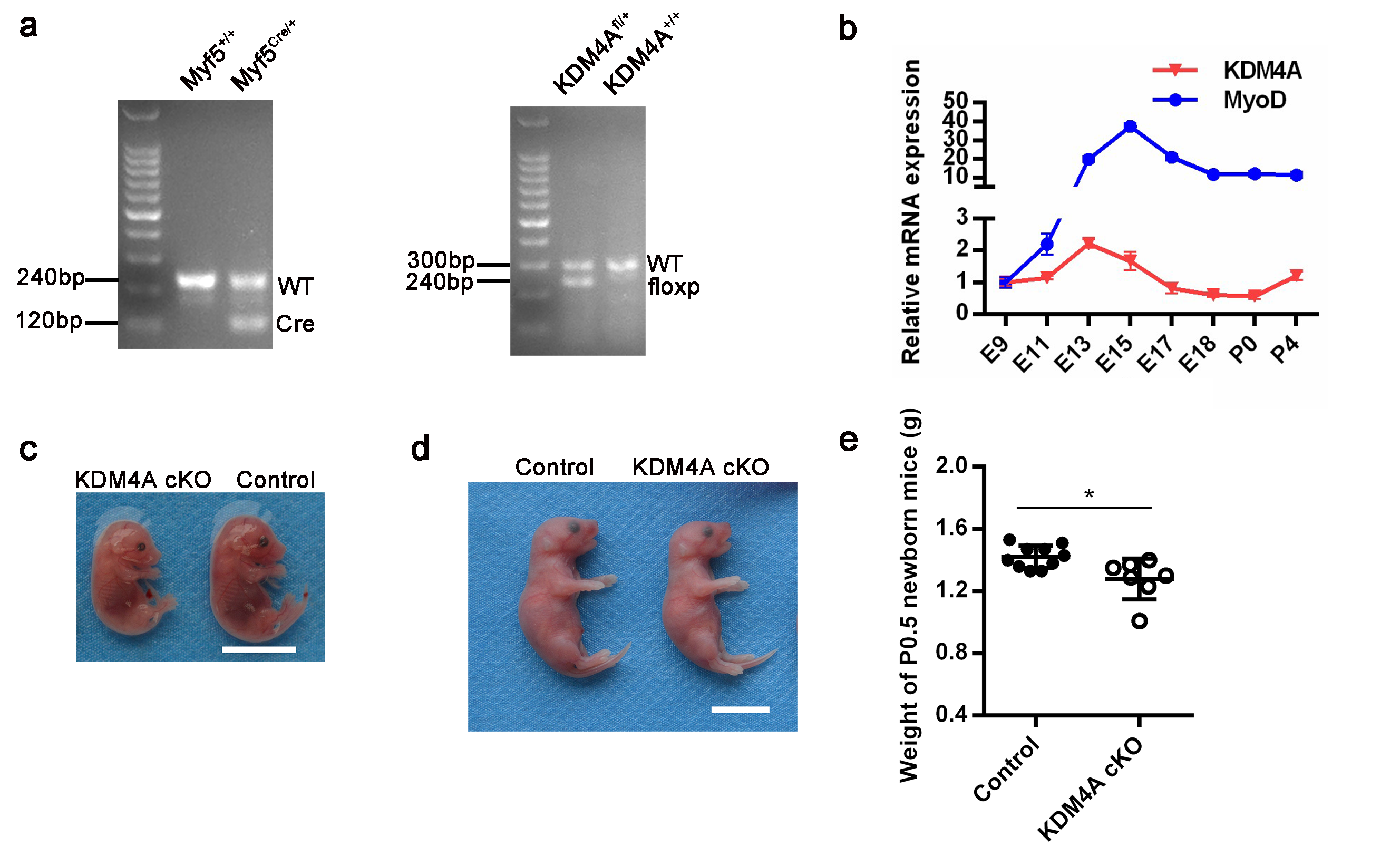

Supplement: Supplementary file 3 — Supplementary Figure 1 [file 41419_2021_3799_MOESM3_ESM.tif]

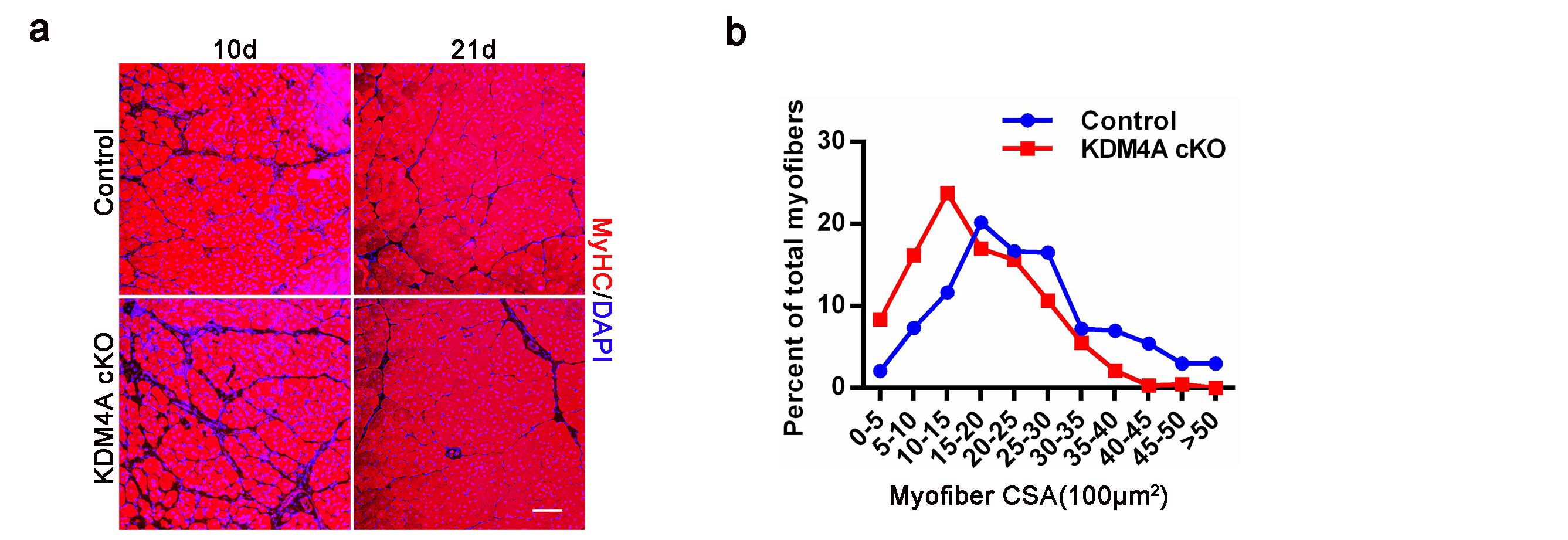

Supplement: Supplementary file 4 — Supplementary Figure 2 [file 41419_2021_3799_MOESM4_ESM.tif]

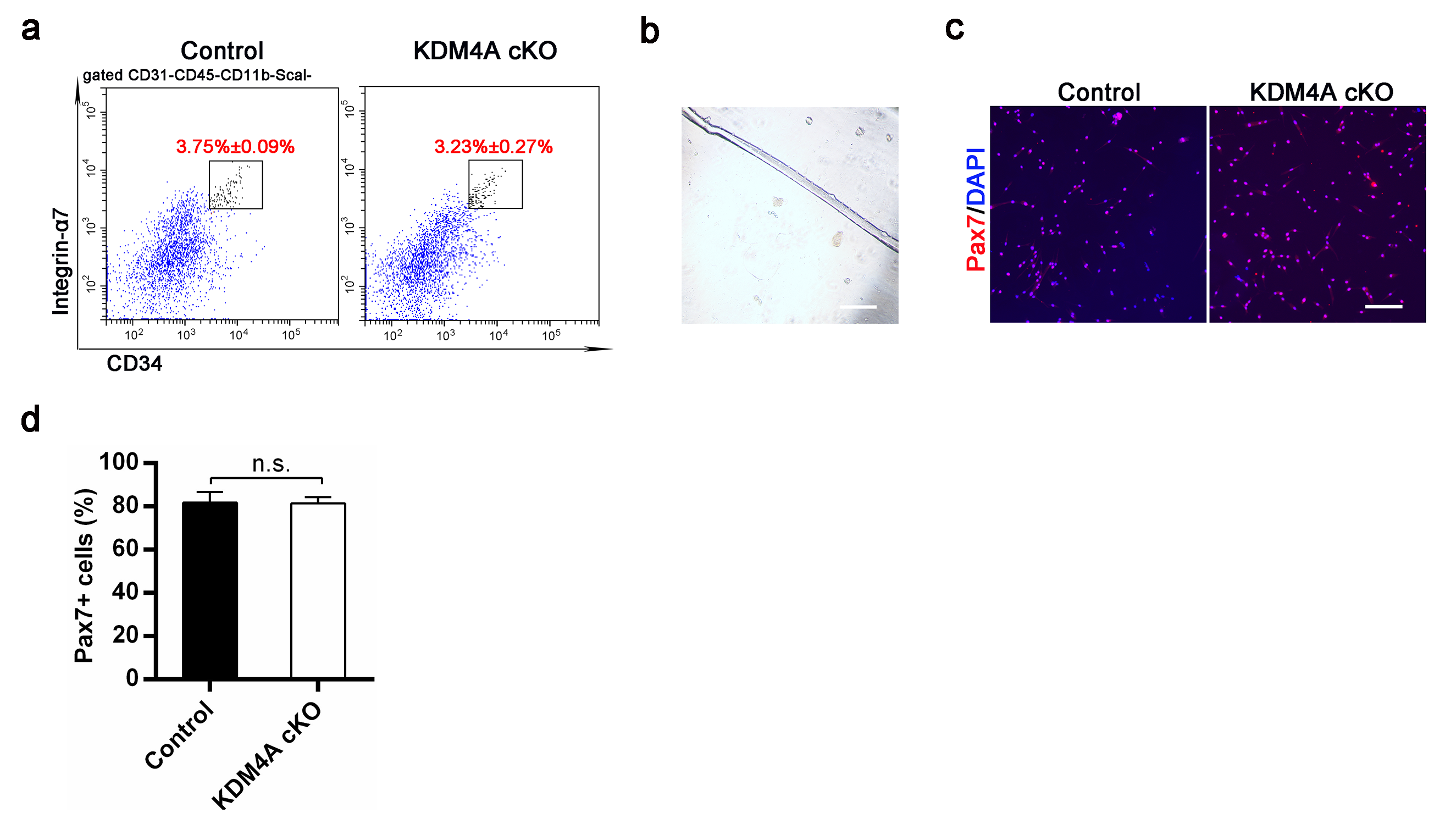

Supplement: Supplementary file 5 — Supplementary Figure 3 [file 41419_2021_3799_MOESM5_ESM.tif]

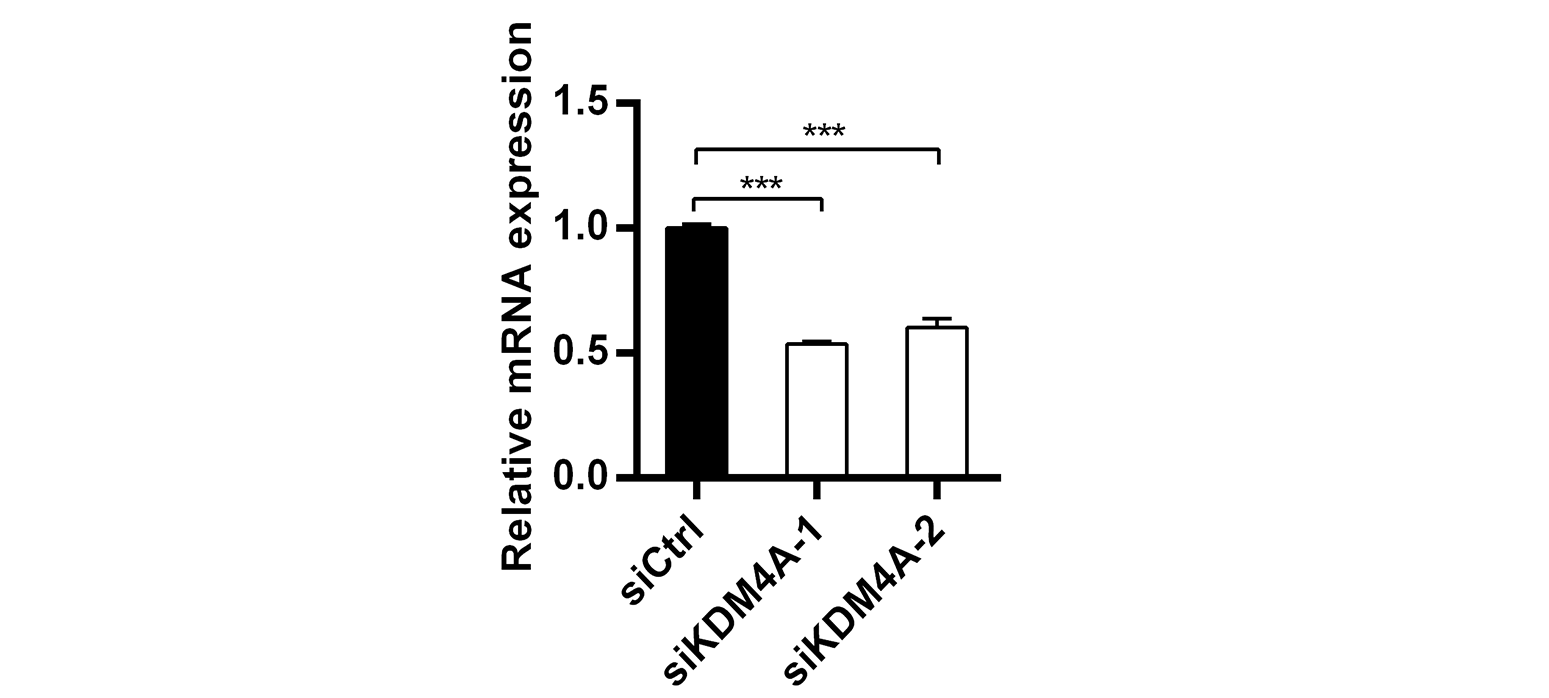

Supplement: Supplementary file 6 — Supplementary Figure 4 [file 41419_2021_3799_MOESM6_ESM.tif]

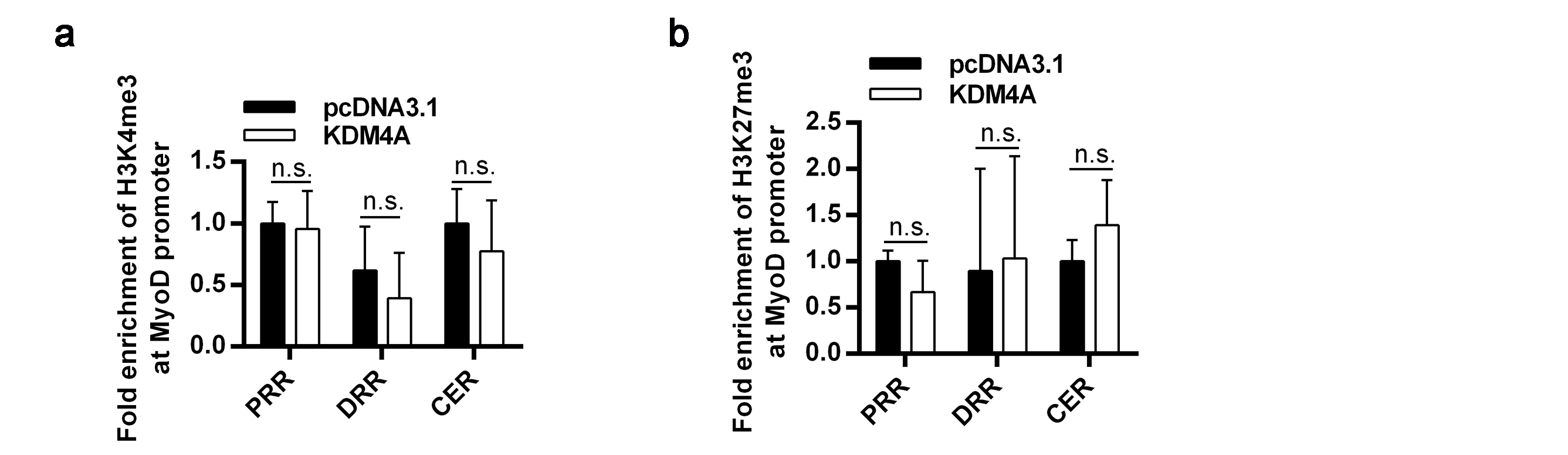

Supplement: Supplementary file 7 — Supplementary Figure 5 [file 41419_2021_3799_MOESM7_ESM.tif]

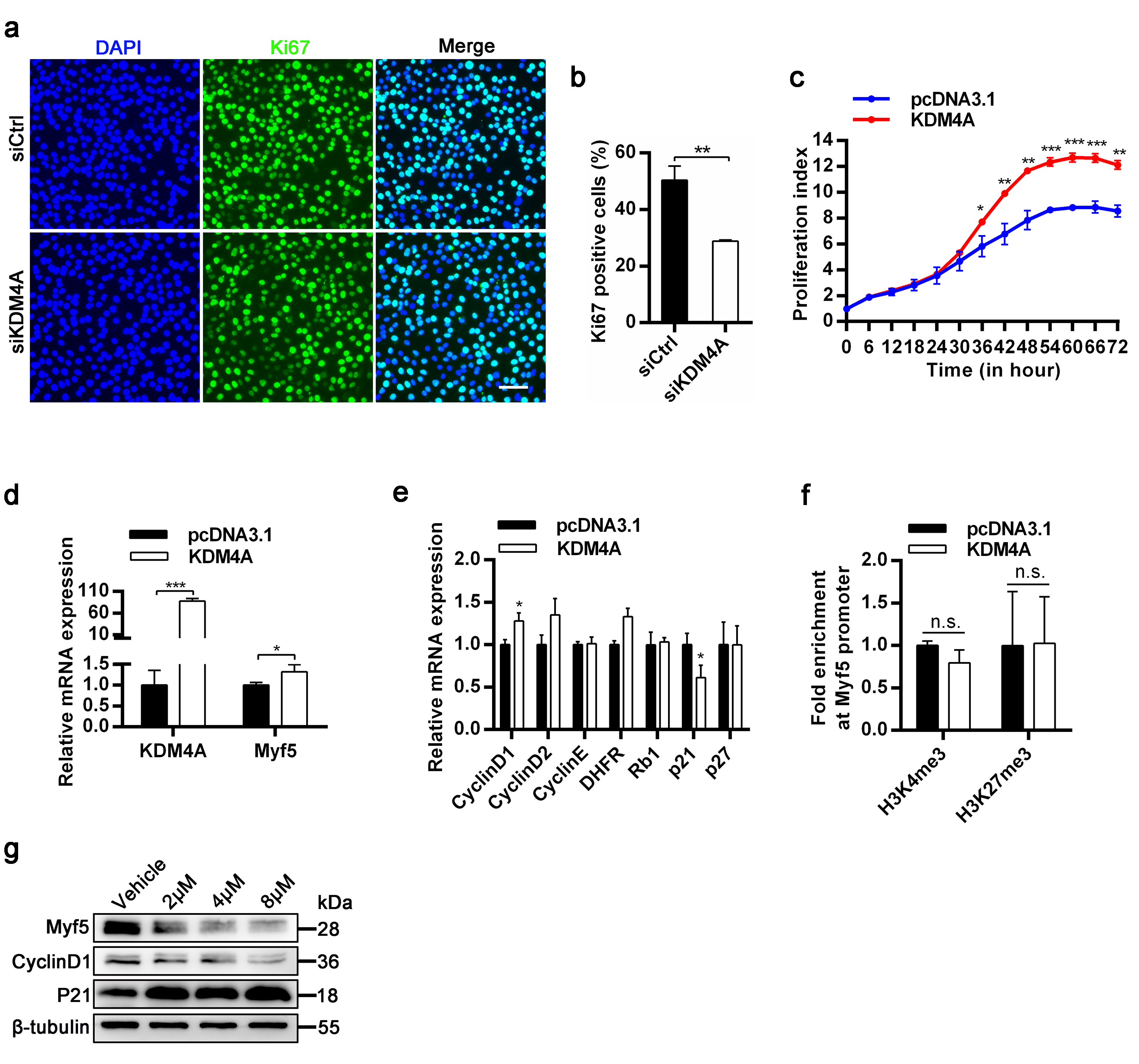

Supplement: Supplementary file 8 — Supplementary Figure 6 [file 41419_2021_3799_MOESM8_ESM.tif]
